# Supplementary material for: Modeling signaling‐dependent pluripotency with Boolean logic to predict cell fate transitions
Source: Mol Syst Biol. 2018 Jan 29;14(1):e7952. doi: 10.15252/msb.20177952 (PMC5787708; doi:10.15252/msb.20177952)
Supplement: Supplementary file 1 — Appendix [file MSB-14-e7952-s001.pdf]

Appendix
"MODELING SIGNALING-DEPENDENT PLURIPOTENCY WITH BOOLEAN LOGIC TO PREDICT CELL FATE TRANSITIONS"

1. **Graph theory-based approximation of gene expression and signaling activity**
   1-1. Random Asynchronous Boolean Simulation for Model Evaluation
   1-2. Defining of SCCs as a sustained PSC population
   1-3. Calculation of probability of each profile in SCC
   1-4. Calculation of sustainability of the SCC-based PSC population
   1-5. Calculation of population-average expression level in PSCs
   1-6. *In silico* subpopulation analysis

2. **Graphical Gaussian Modeling (GGM) to infer GRNs in mESCs**
   2-1. mESC-datasets and the configuration of GGM
   2-2. Assessment of the performance of GRN inference

3. **Reconstruction of Consensus Gene Regulatory Networks in mESCs**
   3-1. Genes considered for model components
   3-2. Signed and directed regulatory edges among the genes
   3-3. Representation of signaling pathway activities and GRNs
   3-4. Consideration of epigenetic-dependent usage of Oct4 enhancer regions
   3-5. Boolean logical functions for the regulatory edges
   3-6. Consensus Gene Regulatory Networks in mESCs

4. **Characterization of PSCs via pluripotency, sustainability and susceptibility**
5. **Simulation of stabilized PSC populations in various culture conditions**
   5-1. *In silico* and *in vitro* manipulation of signal inhibition
   5-2. Simulation for the input conditions mimicking known PSC populations
   5-3. Robustness of calculated population-average gene expression levels
   5-4. Validity of the reconstructed model

1. Graph theory-based approximation of gene expression and signaling activity

1-1. Random asynchronous Boolean simulation for model evaluation

A Boolean network model represents genes with variables that can take on only two possible values: 1 (ON), indicating the gene is sufficiently expressed to drive its downstream regulatory effects, or 0 (OFF) otherwise. A Boolean network with n variables can have 2^n possible states consisting of all combinations of ON/OFF gene expression profiles. We employed asynchronous Boolean simulation (ABS)(Albert and Thakar, 2014; De Cegli et al., 2013), such that individual variables are updated in a random order within each iteration. This strategy accounts for the stochastic nature of gene expression updates(Albert and Thakar, 2014). In ABS, each model state has up to 2^n successor states, including the state itself. Thus, there are up to $2^n \times 2^n = 2^{2n}$ possible transitions between states of the model. The *state transition graph* of a simulated model comprises all the states (nodes) and transitions (directed edges) that were encountered in the simulation. The Boolean network we constructed contains 29 variables; therefore, its state transition graph has a maximum of 5.37×10^8 possible states and 2.88×10^{17} possible transitions between states. Practically, however, the state transition graph limited by the network topology, the update functions, and the presence of stable cycles (e.g. limit cycles and steady states). Because of this, states outside of stable cycles can generally be assumed to be negligible in terms of their frequency of appearance.

Based on this assumption, we employed *random asynchronous Boolean simulation (R-ABS)* to make exploration of the large simulation space more computationally tractable. In R-ABS, simulation trajectories are calculated from a randomly sampled set of initial states. For this study, 700 consecutive iterations from each of 700 random initial states were simulated for each condition. Since independent simulations converged upon similar population-averaged expression probabilities, five independent simulations for each condition were sufficient to yield robust results (see Section 5-3). R-ABS was performed in Python using the BooleanNet package, version 1.2.6 (http://code.google.com/p/booleannet/).

1-2. Strongly connected components as simulated analogues of dynamic heterogeneity in pluripotent stem cell populations

A *strongly connected component (SCC)* is defined as a set of states wherein each state is reachable from every other state in the set (Appendix Figure S1.1). This is reminiscent of observations of dynamic heterogeneity within pluripotent stem cell (PSC) populations, in which individual cells can transition among numerous states that are high or low in their expression of specific pluripotency genes(Filipczyk et al., 2015; Singer et al., 2014). In this study, we assume that model states in an SCC

are analogous to cellular states in a stable yet heterogeneous population of pluripotent stem cells (PSCs). Conversely, since steady states are single states at dead ends in the state transition graph, we assume these reflect relatively homogeneous cell populations, such as differentiated cell states. Note that SCCs are not necessarily closed systems, and SCCs with outgoing transitions were also considered in our analysis. SCCs in a state transition graph can be identified by iterative removal of disconnected states and steady state attractors, which lack either an incoming or outgoing transition edge (Tarjan's algorithm). State transition graph analysis and SCC identification was performed in Python using the NetworkX version 1.2.6.

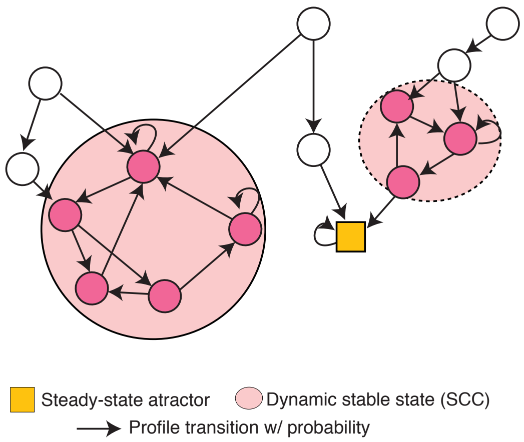

Appendix Figure S1.1. Schematic of SCC identification.

SCCs are defined as the set of nodes (GRN profile) where there is a path between any two nodes.

1-3. Calculation of average expression profile of an SCC

To calculate the average expression profile over all states in an SCC, we first define a *transition matrix* for the SCC. Consider an SCC with N states, identified as $\{s_0, s_1, \ldots s_N\}$, in which each state is a binary vector of the ON/OFF expression level of each gene (ex. 00101). The transition matrix M is an $N \times N$ matrix where each element (m_{ij}) in row i and column j is the probability of transitioning from a source profile s_j to its target profile s_i. For our R-ABS strategy, the transition probability m_{ij} is the frequency of transitions from s_j to s_i divided by the frequency of transitions from s_j to any other state in the SCC ($s_k \in$ SCC). for all simulation trajectories. Note that transitions to states outside the SCC (i.e. to $s_k \notin$ SCC).

Consider a vector $v(0)$, in which each element $v_i(0)$ is the probability that the simulation initially occupies state s_i. After one Boolean update step, the probabilities that the simulation will occupy each state in the SCC can be calculated using the transition matrix, such that $v(1) = Mv(0)$. In general

terms, the probability distribution of each state in the SCC after t Boolean updates is calculated as a Markov process, with $v(t) = Mv(t-1) = M^t v(0)$. Based on the theory of Markov processes, after an indefinite number of Boolean updates (i.e. as $t \rightarrow \infty$), this probability distribution approaches a stable probability distribution, $\tilde{v}$, such that $\tilde{v} = M\tilde{v}$. In other words, $\tilde{v}$ is the principal eigenvector of M, and $\tilde{v}_i$ approximates the biological probability that a given cell in a stable PSC population will have the expression profile corresponding to state s_i.

The average expression profile for an SCC is thus calculated as the expected value of the ON/OFF profiles over all states in the SCC based on each state's stable probability:

$$(AverageExpressionProfileofSCC) = \sum_{i}^{N} \tilde{v}_i s_i$$

1-4. Calculation of sustainability of an SCC

The sustainability of an SCC measures the probability of remaining within the SCC following a Boolean update step, and is a quantitative metric for the intrinsic stability of the SCC. An SCC in which transitions between states within the SCC are much more common than transitions to states outside the SCC has high sustainability (close to 1). This score can be used to estimate the stability of each SCC which reflects the intrinsic stability of the GRN over time in the absence of extrinsic perturbations (see Section 4. "Characterization of PSCs via pluripotency, sustainability and susceptibility"). Mathematically, we define the sustainability score as follows:

$$S_{SCC} = 1 - \sum_{i}^{N} \tilde{v}_i \cdot \left(\sum_{k} m_{ik} \right)$$

where $\tilde{v}_i$ indicates the stable probability of state s_i which has an outgoing transition edge to state $s_k \notin$ SCC. The transition probability from s_k to s_i (m_{ik}) is calculated by the same procedure as above, but considering all successor states of s_k, including those outside of the SCC.

Since outgoing transitions are excluded from the calculation of the stable probabilities, $\tilde{v}$, of an SCC, a high frequency of outgoing edges may lead to overestimation of $\tilde{v}$. Additionally, states in an unstable SCC can easily exit the SCC and transit to another stable state. Thus, sufficiently high sustainability is needed to accurately calculate population-average expression levels and mimic experimentally-observed stable cell states. The size of an SCC is also considered an indicator of the biological accessibility of the SCC-associated population; the larger the SCC, the more accessible it is. Accordingly, we applied thresholds to include only those SCCs with size > 10 and sustainability > 0.7. With these thresholds, only one pluripotency-associated SCC can be found in each of the two standard mESC culture conditions, LIF+Serum (LS) and LIF+2i (2iL).

1-5. Calculation of population-average gene expression levels in simulated PSC conditions

Each state in the state transition graph of a simulated Boolean network is a combination of the binary ON/OFF values for all variables, biologically interpreted as a gene expression profile. Based on the probability and binary levels of each state in an SCC, the average expression level of each variable (g) in that SCC, $p_{g,SCC}$, can be calculated as the weighted sum of all states where the variable is ON:

$$p_{g,SCC} = \sum_{i}^{N} \vartheta_i \cdot \begin{cases} 1, s_i[g] = ON \\ 0, s_i[g] = OFF \end{cases}$$

SCCs were classified to different subtypes based on their average expression levels of the marker genes Cdx2, EpiTFs, Gata6, and Oct4. SCCs with $p_{Cdx2,SCC} > 0.7$ were classified as trophectoderm (TE). Those with $p_{EpiTFs,SCC} > 0.2$ were classified as epiblast (Epi). Those with $p_{Gata6,SCC} > 0.5$ were classified as primitive endoderm (PE). Those with $p_{Oct4,SCC}$ were classified as PSCs. SCCs that co-expressed Cdx2, EpiTFs, and Gata6 were classified as mesendoderm (ME). Note that SCCs with coinciding high levels of Oct4 and EpiTFs were assumed to be in the "primed" PSC state, whereas those with high levels of Oct4 alone were assumed to be in the "naïve" PSC state.

Large dynamic stable states of PSCs are more likely to dominate the total cell population over time and thus will become a larger determinant of the population-average gene expression levels. Thus, population-average gene expression levels are defined as a function of both size and sustainability of each SCC:

$$p_{g,Population} = \frac{\left(\sum_{SCC}^{R} p_{g,SCC} \cdot n_{SCC} \cdot S_{SCC}\right)}{\sum_{SCC}^{R} n_{SCC}}$$

where R is the set of all SCCs found under the given condition meeting the above-mentioned thresholds. An example of this calculation is shown in Appendix Figure S1.2.

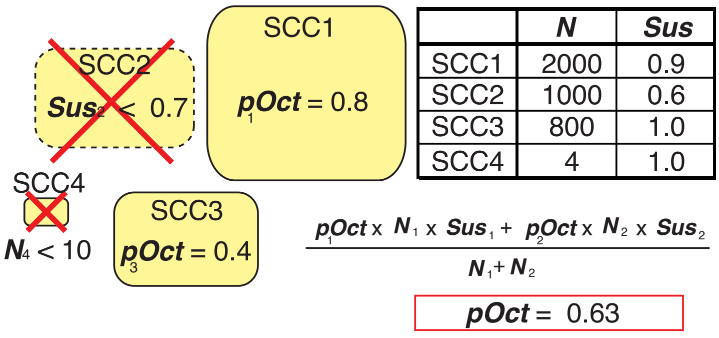

Appendix Figure S1.2. Calculation example of population-average gene expression levels.
This figure assumes that four distinct SCCs are found from the given input. SCC#2 and SCC#4 are excluded from the population-average calculation due to low sustainability and small number of nodes, respectively. The rest of the SCCs (SCC#1 and SCC#3) are considered in the final population-average expression level.

1-6. *In silico* subpopulation analysis

Unlike steady states and completely closed SCCs (sustainability = 1), states within non-closed SCCs (sustainability < 1) can transit to other SCCs and steady states. The probability of transitioning between distinct SCCs, and thus between distinct PSC subpopulations, can be calculated from the state transition graph. Specifically, the transition probability from one SCC (A) to another (B), $T_{A\to B}$, is the product of the probability of each transition for all possible trajectories from A to B.

$$T_{A\to B} = \sum_{i}^{N} \vartheta_i \cdot \left(\sum_{L_i} \left(\prod_{j} k_j \right) \right)$$

where ϑ_i is the stable probability of state s_i in A, L_i is the set of possible trajectories from s_i to any state in B after eliminating all cycles, and k_j is the probability of each transition along the trajectory. It follows that if all transitions from A collapse into B, then $T_{A\to B} = 1$.

2. Graphical Gaussian Modeling (GGM) to infer GRNs in mESCs

2-1. mESC-datasets and the configuration of GGM

Conventionally, overexpression and knockdown/knock-in studies of genes of interest and subsequent biochemical experiments have been performed to identify regulatory networks within specific conditions in mESCs. High-throughput genome binding studies such as chromatin immuno-precipitation, coupled with massively parallel short-tag-based sequencing have also been used to infer important regulatory edges in the relevant GRN. Although the resulting data exhibits large variations across cell lines and between datasets from different labs, phenotypic responses to *in vitro* manipulation of signaling cues are reasonably robust, leading us to argue that the individual regulatory "rules" underlying GRN connectivity are conserved among cell lines across environmental situations. Thus, inference of GRNs using a large collection of unbiased expression data should effectively yield consensus network topologies. A number of computational reverse engineering approaches have been developed for reconstructing cellular transcription networks from gene expression data, including those of mESCs(De Cegli et al., 2013). One method employed in some of these approaches is partial correlation (pcor), which infers direct dependencies among pairs of genes x and y by removing the effects of a third gene z. Given the Pearson correlation value of the different combinations of the three genes (r_{xy}, r_{xz}, r_{yz}), the partial correlation of x and y is calculated as:

$$r_{xy,z} = \frac{r_{xy} - r_{xz} \cdot r_{yz}}{\sqrt{1 - {r_{xz}}^2}\sqrt{1 - {r_{yz}}^2}}$$

We collected 1,295 Affymetrix Mouse 430 2.0 Array microarray datasets on mESCs from the Gene Expression Omnibus (GEO) database at the US National Center for Biotechnology

Information (NCBI) and ArrayExpress at the European Bioinformatics Institute (EBI) (See Appendix of this Supplementary Note). It is important to use the expression data from the same platform because the sensitivities of probes for a certain target region are not uniform over different platforms. All microarray datasets were collected regardless of cell line, manipulation type (e.g. knock-in/out), whether experiments were timecourses or dose responses. We did so with the rationale that if there are direct and robust regulations from gene x to gene y, the positive or negative correlation between x and y will be conserved in all expression data regardless of perturbation, lab, or cell line.

Graphical Gaussian Modeling (GGM) was used to infer partial correlations between gene pairs (Appendix Figure S2.1). GGM permits analysis of complex networks involving a large number of nodes (large network size) and multiple edges between nodes (high network connectivity), as is the case in mammalian GRNs(Ma et al., 2007). Data for all 45,101 probe sets were first normalized with a quantile algorithm in the 'limma' package of R/Bioconductor. The probe sets were then converted into 13,879 individual genes by taking mean values of probes with the same gene annotation.

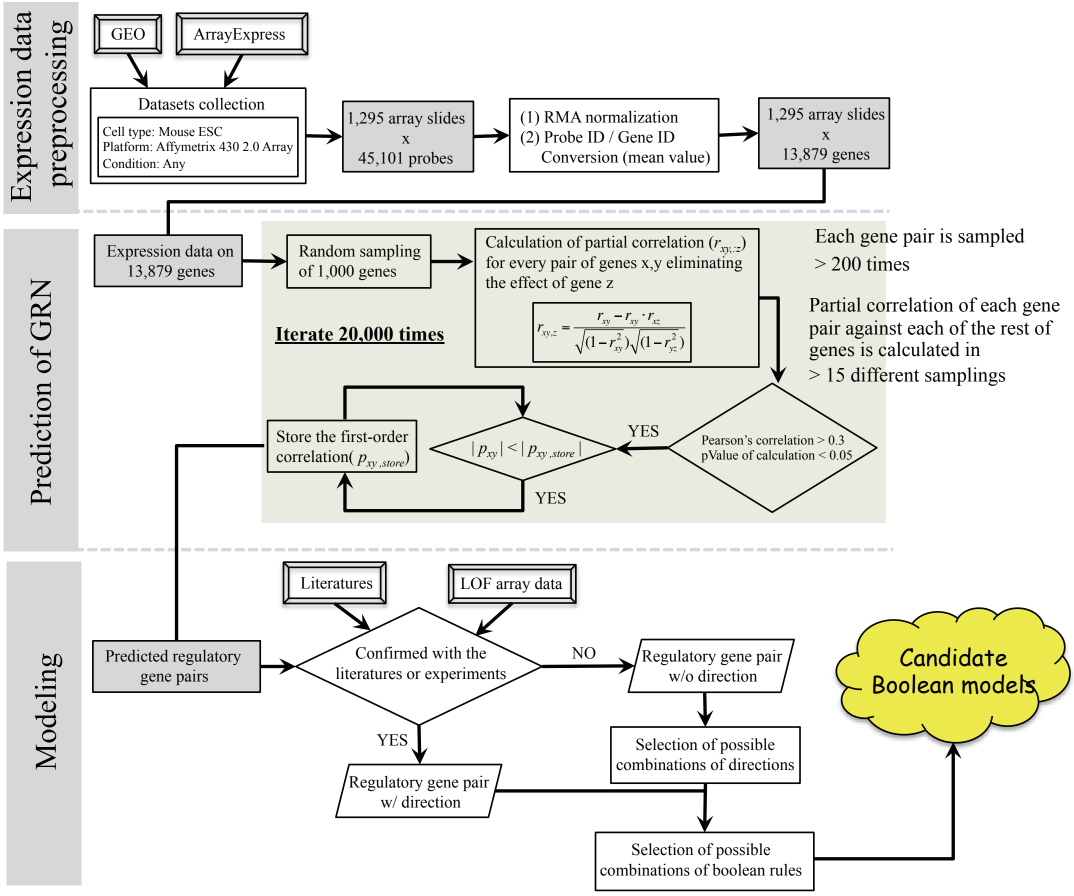

Appendix Figure S2.1. Flow-chart of network reconstruction based on Graphic Gaussian Modeling.

In the GGM network construction, 1,000 genes were randomly sampled to estimate partial correlations in 20,000 total iterations. It is estimated that, over all iterations, each gene pair is sampled 200 times on average. Significant partial correlations (pcor) for each iteration were selected if they met two conditions: (1) absolute Pearson correlation value of the gene pair wasgreater than 0.3 and (2) the p-value was less than 0.05. The final pcor value was estimated by taking the lowest pcor value for each gene pair after completion of all iterations. The calculation of pcor in each iteration was performed using the R/GeneNet package and the whole GGM algorithm, including the random sampling of genes, was scripted using Python.

2-2. Assessment of the performance of GGM-based GRN inference

We first tested for batch effects in the collected expression datasets. The datasets were divided randomly into two groups with constraints that array samples from the same studies were kept in the same group and that the number of total samples within each group did not exceed 500. The gene-to-gene relationships were estimated within each group using Context Likelihood of Relatedness (CLR). The output of CLR was then binarized based on various thresholds ranging from the 70th to 98th percentiles. Using the binarized CLR output of one of the two groups as a template, the area under the Receiver Operating Characteristic curves (AUC-ROC) was calculated between two groups. The average AUC-ROC for 1,000 random samples of the experimental datasets was maximized (0.82) at the 90th percentile threshold (Appendix Figure S2.2a). The low standard deviation (0.025) indicates that batch effects among the collected datasets are negligible for inference of the GRN. To further characterize the robustness of GRN inference to the input microarray data, we compared the gene-gene relationships inferred by CLR using the full set of microarray data (1,295 samples) to those inferred from variously-sized partial datasets from of randomly selected microarray profiles (Appendix Figure S2.2b). The results indicated that a relatively small number of samples (>200) are feasible to replicate the full dataset showing correlation coefficients as high as 0.9, and sufficient for robust GRN inference.

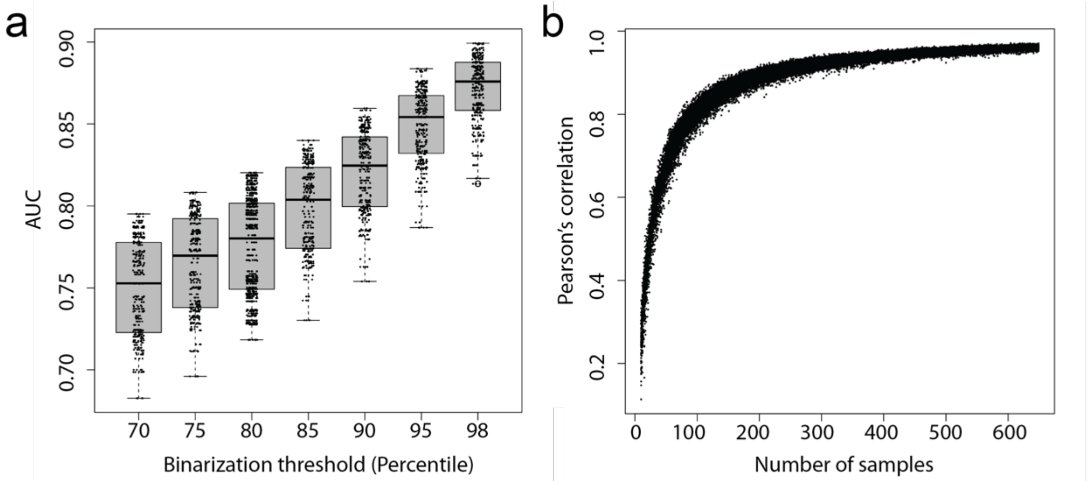

Appendix Figure S2.2. Assessment of the robustness of the expression dataset.

a. Small dot indicates the result in each sampling where experimental samples are divided into two groups. **b.** Small dots represent Pearson's correlation coefficient between gene-to-gene relationships inferred by CLR algorithm based on either the full (1,295) dataset or the partial dataset where the number of samples is indicated in x-axis.

Next, to assess the reliability of our network inference approach, we compared the predicted regulations against the ESCAPE database (Embryonic Stem Cell Atlas from Pluripotency Evidence; www.maayanlab.net/ESCAPE). ESCAPE is a collection of directed gene-to-gene interactions supported by published expression datasets, supplemented by loss-of-function (LOF) or gain-of-function (GOF) analyses of various transcription factors (TFs) and results from ChIP-chip/ChIP-seq studies. We confirmed that high-scoring inferred interactions could be predicted more accurately than random selection by plotting a Precision-Recall graph (Appendix Figure S2.3a). To account for the lack of directionality (i.e. positive or negative), the scores of inferred interactions from the experimentally tested genes were converted into absolute values. The interactions that were found as significant in any of the experiments (i.e. LOF, GOF or ChIP-chip/ChIP-seq) were considered as biologically-observed interactions.

We then compared the performance of our GRN inference approach against other well-established algorithms including Algorithm for the Reconstruction of Accurate Cellular Networks (ARACNe(Margolin et al., 2006a, 2006b)), Context Likelihood of Relatedness (CLR(Faith et al., 2007)), TwixTrix(Qi and Michoel, 2012) and simple Pearson's correlations (PPC) using the same expression datasets. Our GGM-based approach had the best performance among all tested algorithms when the ROC curves of the top 10,000 inferred interactions of each algorithm were compared against ESCAPE data (Appendix Figure S2.3b). Interestingly, our approach, in contrast to all other algorithms, detected the experimentally-verified negative relationship between Nanog and Gata6 (highest negative correlation for Nanog).

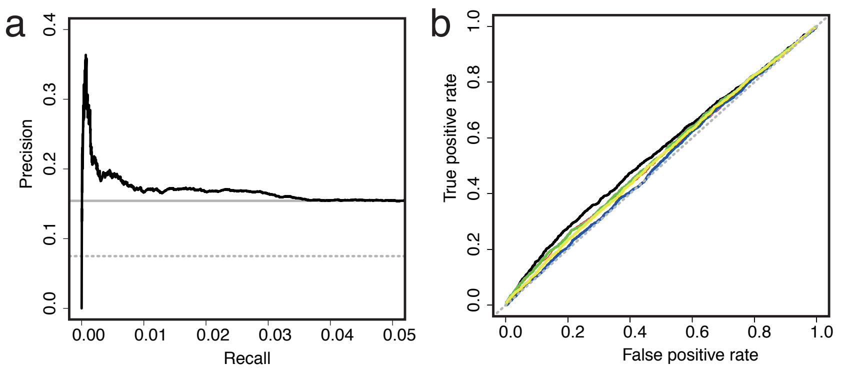

Appendix Figure S2.3. Performance of GGM-based GRN inference.
a. Precision-Recall graph of predicted interactions against ESCAPE dataset. Random value of precision is indicated with a dotted line (calculated by taking the total of 107,663 interactions found in ESCAPE versus possible interactions among 13,879 genes and 107 experimentally tested genes excluding self-regulating interactions). The gray line indicates the average precision value. b. ROC curve for each different algorithm. ROC curve shows the true positive rate (TPR) and the false positive rate (FPR). Our GGM-based approach, ARACNe, CLR, TXIXTRIX and PCC are indicated with black, red, yellow, green, and blue lines, respectively. Random value (TPR=FPR) is shown with dotted line. Top 10,000 inferred interactions were considered.

For the 29 genes included in the model, the precision of the GGM-based inference approach (black) was higher than that of the non-iterative GGM (orange) approach (i.e. GGM for all genes), achieving a lower recall range with higher precision (Appendix Figure S2.4). However, the accuracy was still close to random (Accuracy: 0.52, Sensitivity: 0.45, Specificity: 0.69 at the highest accuracy). This may be because our approach only keeps interactions above the specified statistical threshold, or because of limitations in the collected experimental datasets, including biological noise and the biased selection of genes. Indeed, even for well-analyzed biological systems (*E. coli*, yeast, etc.), individual GRN inference algorithms and community prediction are only marginally more accurate than random selection.(Marbach et al., 2012; Hase et al., 2013).

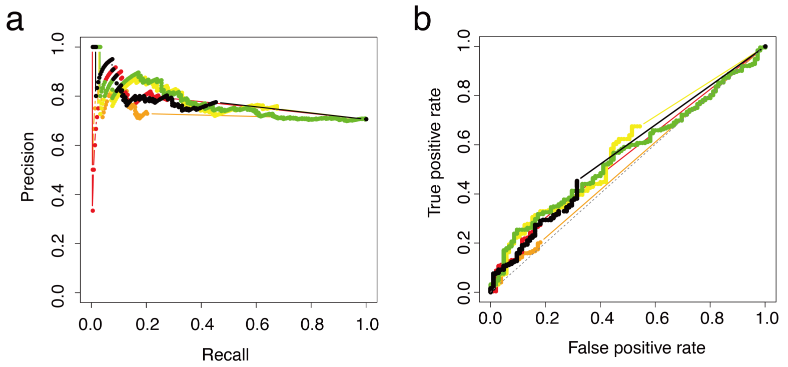

Appendix Figure S2.4. Performance of GGM-based GRN inference for the genes included in the model.
a. Precision-Recall graph of predicted interactions against the ESCAPE dataset of the gene pairs each of which is included in the model. Our GGM-based approach, ARACNe, CLR, TXIXTRIX and regular GGM are indicated with black, red, yellow, green, and orange lines, respectively. **b**. ROC curve for the set of algorithms in **a**.

To further investigate the generalizability of our GRN inference approach, we applied it to the transcriptome datasets of *E. coli* for the DREAM5 network inference challenge(Marbach et al., 2012) and compared the results with the reported benchmarks of other approaches. Interestingly, our GGM-based approach scored higher for area under the precision-recall curve than the community prediction (Appendix Fig. S2.5), which indicates the robustness of our approach.

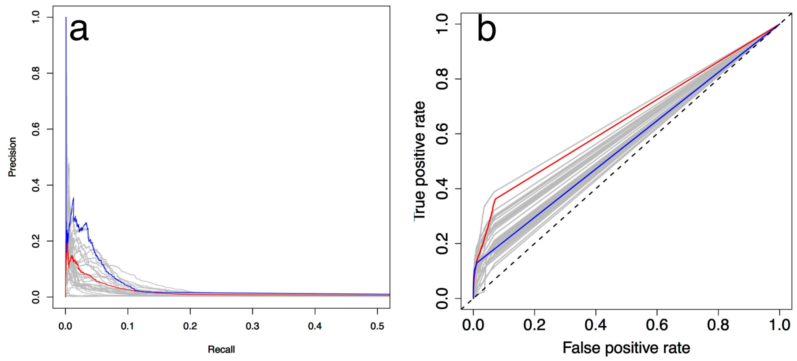

Appendix Figure S2.5. Performance of GGM-based GRN inference on e.coli datasets of DREAM5 network inference challenge.

a. Precision-Recall curves and **b**. ROC curves for the individual methods (gray), the integrated community predictions for them (red), and the predictions with our GGM-based approach (blue) against the selected gold standards with strong experimental support. In the prediction, 1,000 genes out of 4,297 genes were randomly sampled to estimate partial correlations for each 20,000 iteration. The potential regulatory relationships among 304 transcription factors and 4,297 genes were considered in the plots.

3. Reconstruction of Consensus Gene Regulatory Networks in mESCs

3-1. Genes considered in the model

Based on knowledge from literature and the inferred gene pairs with positive/negative correlations, the model gene list was condensed to 29 genes. We first focused on the central pluripotency genes (*Oct4*, *Sox2* and *Nanog* [OSN]), genes supporting somatic cell reprogramming(Takahashi and Yamanaka, 2006) (*Klf4* and *Myc*), other known pluripotency regulators around OSN and naïve-PSC specific TFs(De Los Angeles et al., 2015; Heng et al., 2010; Loh et al., 2006; Masui et al., 2008; Varlakhanova et al., 2010) (*Esrrb*, *Tbx3*, *Gbx2*, *Lrh1*, *Jarid2*, *Klf2*, *Mycn*, *Pecam1* and *Rex1*). Key lineage specifiers (*Tcf3*, *Gata6*, *Gcnf* and *Cdx2*) which contribute to the exit of pluripotency and which participate in negative regulatory loops with OSN were also included in the model(Chickarmane and Peterson, 2008; Tam et al., 2008). For computational efficiency, we aggregated EpiSC-enriched genes such as Brachyury (*T*), *Fgf5*, *Eomes* and *Otx2* into a single component in the model termed EpiSC-enriched transcription factors (EpiTFs). We also considered an epigenetic aspect of the GRN where DNA methylation status alters enhancer sites of *Oct4* by the availability of *Dnmt3b* and *Klf2* (see section 3-4 for details). The gene list was further extended to include genes which are predicted to be highly correlated with the previously noted core pluripotency genes (*Pitx2* and *Dusp6*).

We also included components of key signaling pathway activities: canonical WNT, BMP4, Activin A/Nodal, and FGF signaling. These included receptors (*Fgfr2*), cytokine-coding genes (*Bmp4*, *Nodal*, *Fgf4*), and inhibitors (*Lefty1* – negative regulator for Activin A/Nodal signaling(Thisse and Thisse, 1999), *Smad6* – negative regulator of BMP4 signaling(Hata et al., 1998), and *Smad7* – negative regulator for both Activin A/Nodal and BMP4 signaling(Yan et al., 2009). Note that these genes are also predicted to be highly correlated with the core pluripotency genes). Note that *Cdx2* is considered an inhibitor of WNT signal activity, based on work done by Liu and colleagues(Liu et al., 2012) as well as the observed high positive correlation between *Cdx2* and *Dkk1* (pcor=0.043)(Lewis et al., 2008). *Pitx2* and Dusp6 were also included as both downstream targets of signals (WNT- and Activin A/Nodal for *Pitx2*, and FGF for *Dusp6*) and as regulators of core TFs (e.g. *Klf2*).

Note that other TFs reported to have important roles for pluripotency maintenance such as *Tcfcp2l1* and *Klf5* were not included due to significant overlaps in correlated gene partners with *Esrrb* and *Klf4* (*p-values* $< 10^{-270}$ for positively correlated genes), respectively.

3-2. Signed and directed regulatory edges among the genes

Based on the inferred GRN, the links among 29 genes were identified as highly correlated gene pairs (as high as ±0.03). The directionality of regulation for each pair was determined by experimental evidence, either by curating published low-throughput experiments or by scoring high-throughput data from ESCAPE with weighted majority voting:

$$E_{i,j} = \lceil\frac{N_{LOF}(i,j)}{T(i)_{LOF}}\rceil + 0.3 \cdot \lceil\frac{N_{GOF}(i,j)}{T(i)_{GOF}}\rceil + 0.5 \cdot \left|\frac{N_{Chip}(i,j)}{T(i)_{Chip}}\right|$$

where $N(i,j)$ is the number of experiments which show positive effects (activation; positive values) or negative effects (inhibition; negative values) from gene i to gene j, according to LOF or GOF, or ChIP-seq/-chip studies in the ESCAPE database, and $T(i,j)$ is the total number of experiments testing the effects of gene i. The positive/negative directions ($D(i,j)$) were evaluated by the summation of $N_{LOF}(i,j)$ and $N_{GOF}(i,j)$.

Our GGM analysis suggested interesting interplays between core pluripotency TFs and signaling activities. For example, *Nanog* and *Oct4* were predicted to be negative regulators for *Fgfr2*, which codes for a receptor of FGF signaling. Both *Nanog* and *Oct4* have been shown to bind the *Fgfr2* promoter by multiple genome-binding studies and confirmed to downregulate *Fgfr2* by loss of function (LOF) studies.

As shown in Appendix Table S1a, the directionalities of 76 out of 86 inferred gene-gene regulatory links were determined in this evidence-based step. The directionalities of two gene pairs (*Lefty1-Activin A/Nodal* and *Nr5a2(Lrh1)-Oct4*) were modified based on literatures(Gu et al., 2005; Guo and Smith, 2010; Heng et al., 2010). *Lefty1* is a known inhibitor and direct target of Nodal/Activin A signaling(Lee et al., 2011). Our GRN inference may have captured the latter relationship as a positive correlation. Following this step, there remained 10 gene pairs with undetermined directionalities that are highlighted in gray in Appendix Table S1. The directionalities of these 10 edges were eventually determined with best-fit model selection by calculation of difference in population-average gene expression levels among the possible models and the single cell expression data of mESCs in LS(Kolodziejczyk et al., 2015; MacArthur et al., 2012) (Section 3-6).

a.

Source Gene	Target Gene	Actions on directionality	Evidences (PubMed ID)	+/-	GGM	$D_{i,j}$	$E_{i,j}$	LOF	GOF	ChIP
Tbx3	Smad7	Determined	Target not TF	+	0.099	0	0.0		NE	
Klf4	Smad7	Determined	Target not TF/ **21501463;**	+	0.080	-1	0.5		1-	1
Klf4	Tbx3	Determined	Evaluation by ESCAPE	+	0.080	2	1.1	1+	1+	2
EpiTF(Otx2)	Dnmt3b	Determined	Target not TF/ Evaluation by ESCAPE	+	0.076	1	0.3	NE	1+	NE
Nanog	Esrrb	Defined / Determined	Evaluation by ESCAPE	+	0.072	2	1.2	2++		2
Klf2	Esrrb	Determined	**18264089;**	+	0.071	NE	0.0	NE	NE	
**Esrrb**	**Klf4**	Defined / Determined	Evaluation by ESCAPE	+	0.070	2	1.5	2++	NE	1
**Klf4**	**Esrrb**	Defined / Determined	Evaluation by ESCAPE	+	0.070	1	0.5	1+		
Lefty1	Nodal/ActivinA	Defined	Target not TF/ **10518210;**	–	0.069	NE	NE	NE	NE	NE
Gbx2	Myc	Predicted	---	+	0.065	NE	NE	NE	NE	NE
Myc	Bmp4	Determined	Target not TF/ 10457277;16010442	+	0.062	0	0.0	NE		1
Sox2	Klf4	Determined	Evaluation by ESCAPE	+	0.062	2	1.0	2++		
Esrrb	Smad7	Determined	Target not TF/ Evaluation by ESCAPE	+	0.054	0	0.5		NE	1
Dusp6	EpiTF(Eomes,Fgf5,Otx2)	Predicted	---	+	0.052	NE	0.0	NE	NE	NE
Gata6	Smad6	Determined	Target not TF	+	0.051	NE	NE	NE	NE	NE
Tbx3	Bmp4	Determined	Target not TF/ Evaluation by ESCAPE	+	0.051	1	1.5	1+	NE	1
Pitx2	EpiTF(Eomes,Fgf5)	Predicted	---	+	0.050	NE	NE	NE	NE	NE
Mycn	Klf2	Determined	**19495417;**	+	0.048	0	0.0	NE		
Sox2	Klf2	Determined	Evaluation by ESCAPE	+	0.047	2	1.5	2++		4
Nr6a1	Fgfr2	Determined	Target not TF	+	0.046	NE	NE	NE	NE	NE
**Klf2**	**Klf4**	Defined / Determined	Evaluation by ESCAPE	+	0.044	NE	0.0	NE	NE	1
**Klf4**	**Klf2**	Defined / Determined	Evaluation by ESCAPE	+	0.044	0	1.3	1+	1-	3
Gbx2	Bmp4	Determined	Target not TF	+	0.043	NE	NE	NE	NE	NE
Mycn	Dnmt3b	Determined	Target not TF	+	0.043	0	0.0	NE		
Nanog	Smad7	Determined	Target not TF/ Evaluation by ESCAPE	+	0.043	0	0.3			3
Cdx2	Fgfr2	Determined	Target not TF/ Evaluation by ESCAPE	+	0.042	1	0.3	NE	1+	
Pou5f1	Nodal/ActivinA	Determined	Target not TF/ Evaluation by ESCAPE	+	0.041	2	1.1	2++		4
**Tbx3**	**Nanog**	Defined / Determined	**19571885; 20734354;**	+	0.040	0	0.0		NE	
**Nanog**	**Tbx3**	Defined / Determined	Evaluation by ESCAPE	+	0.040	0	0.4			4
Tbx3	Nr5a2	Determined	Evaluation by ESCAPE	+	0.039	0	0.5			1
Tcf3	Dnmt3b	Predicted (Posi/Nega)	Target not TF/ Evaluation by ESCAPE	+	0.037	-1	0.8		1-	3
**Nanog**	**Pou5f1**	Defined / Determined	Evaluation by ESCAPE	+	0.036	2	1.3	1+	1+	5
**Pou5f1**	**Nanog**	Defined / Determined	Evaluation by ESCAPE	+	0.036	1	1.5	2++	1-	5
Cdx2	Nr6a1	Determined	**18941526;**	+	0.035	0	0.0	NE		
Nr5a2	Nanog	Determined	**20096661;20734354;**	+	0.035	0	0.0			NE
**Nanog**	**Sox2**	Defined / Determined	Evaluation by ESCAPE	+	0.035	1	1.0	1+		5
**Sox2**	**Nanog**	Defined / Determined	Evaluation by ESCAPE	+	0.035	0	0.4			3
Esrrb	Tbx3	Determined	Evaluation by ESCAPE	+	0.034	1	1.0	1+	NE	1
**Klf4**	**Nanog**	Defined / Determined	Evaluation by ESCAPE	+	0.033	1	1.0	1+		3
**Nanog**	**Klf4**	Defined / Determined	Evaluation by ESCAPE	+	0.033	1	0.6	1+		1
Klf4	Jarid2	Determined	Evaluation by ESCAPE	+	0.032	0	0.5			3
Pou5f1	Mycn	Determined	Evaluation by ESCAPE	+	0.032	3	1.3	3+++		3
Jarid2	Gbx2	Determined	Evaluation by ESCAPE	+	0.032	0	0.5			2
Sox2	Mycn	Determined	Evaluation by ESCAPE	+	0.031	1	1.0	1+		4
Mycn	Lefty1	Determined	Evaluation by ESCAPE	+	0.031	1	0.8	NE	1+	1
Pou5f1	Lefty1	Determined	Evaluation by ESCAPE	+	0.031	3	1.5	3+++		5
Sox2	Nr5a2	Determined	Evaluation by ESCAPE	+	0.031	2	1.1	2++		1
Esrrb	Fgf4	Determined	Evaluation by ESCAPE	+	0.031	2	1.5	2++	NE	1
Klf4	Nr5a2	Determined	Evaluation by ESCAPE	+	0.031	2	0.8	1+	1+	
Pou5f1	Smad7	Determined	Target not TF/ Evaluation by ESCAPE	+	0.031	1	0.6	1+		3
Esrrb	Gbx2	Determined	Evaluation by ESCAPE	+	0.031	1	1.0	1+	NE	1
Nanog	Fgf4	Determined	Evaluation by ESCAPE	+	0.031	2	1.3	2++		3
Pou5f1	Jarid2	Determined	Evaluation by ESCAPE	+	0.031	1	0.7	1+		4
Klf2	Pou5f1	Determined	**20875146;**	+	0.031	NE	0.0	NE	NE	
Sox2	Lefty1	Determined	**16954384 (positive regulation)**	+	0.031	-2	1.2	1-	1-	3
**Klf4**	**Pou5f1**	Defined / Determined	Evaluation by ESCAPE	+	0.030	1	0.8		1+	3
**Pou5f1**	**Klf4**	Defined / Determined	Evaluation by ESCAPE	+	0.030	2	1.3	3+++	1-	
**Nr5a2**	**Pou5f1**	Defined / Determined	**15831456;20096661;**	+	-0.032	1	0.3		1+	NE
**Pou5f1**	**Nr5a2**	Defined / Determined	**18522731;19884255;24332857;**	+	-0.032	1	1.1	2++	1-	1
Sox2	Nodal/ActivinA	Determined	Target not TF/ Evaluation by ESCAPE	-	-0.033	-2	1.2	1-	1-	3
Jarid2	Pitx2	Determined	Evaluation by ESCAPE	-	-0.034	0	0.5			2
Sox2	Pitx2	Determined	Evaluation by ESCAPE	-	-0.036	-3	1.4	2--	1-	1
EpiTF(Eomes)	Nr6a1	Predicted	---	-	-0.0416	0	0.0	NE		NE
Fgf4	Gata6	Predicted	---	-	-0.045	NE	NE	NE	NE	NE
Pou5f1	Smad6	Determined	Target not TF/ Evaluation by ESCAPE	-	-0.048	-2	0.8	2--		1
Sox2	EpiTF(Eomes)	Determined	Evaluation by ESCAPE	-	-0.049	-1	1.3	2--	1+	
Gbx2	Nodal/ActivinA	Determined	Target not TF	-	-0.050	0	NE	NE	NE	NE
Jarid2	Smad7	Determined	Target not TF/ Evaluation by ESCAPE	-	-0.052	-1	1.0	1-		
Klf2	Gata6	Predicted	---	-	-0.053	NE	0.0	NE	NE	
Pitx2	Klf2	Predicted	---	-	-0.053	NE	NE	NE	NE	NE
Gbx2	Smad7	Determined	Target not TF	-	-0.056	NE	NE	NE	NE	NE
Pou5f1	Fgfr2	Determined	Target not TF/ Evaluation by ESCAPE	-	-0.059	-2	1.0	2--		3
Fgf4	Lefty1	Predicted	---	-	-0.059	NE	NE	NE	NE	NE
Dusp6	Klf2	Predicted	---	-	-0.059	NE	0.0	NE	NE	NE
Jarid2	Lefty1	Determined	Evaluation by ESCAPE	-	-0.060	-1	1.0	1-		
EpiTF(Fgf5, Otx2)	Esrrb	Determined	Evaluation by ESCAPE	-	-0.062	-1	0.3	NE	1-	NE
Nanog	Mycn	Determined	Evaluation by ESCAPE	-	-0.064	-1	0.7		1-	4
**Gata6**	**Nanog**	Defined / Determined	**11937486; 17605826;**	-	-0.074	NE	NE	NE	NE	NE
**Nanog**	**Gata6**	Defined / Determined	**15983365;16456133;**	-	-0.074	0	0.8	1-	1+	
Tbx3	Gbx2	Determined	Evaluation by ESCAPE	-	-0.080	-1	1.0	1-	NE	
Nanog	Fgfr2	Determined	Target not TF/ Evaluation by ESCAPE	-	-0.082	-2	1.2	1-	1-	4
Cdx2	Dnmt3b	Determined	Target not TF/ Evaluation by ESCAPE	-	-0.086	-1	0.3	NE	1-	
**EpiTF (Otx2)**	**Klf4**	Determined	Evaluation by ESCAPE	-	-0.102	-1	0.3	NE	1-	NE
**Klf4**	**EpiTF (Otx2)**	Determined	Evaluation by ESCAPE	-	-0.102	-3	1.3	2--	1-	
Klf4	Dnmt3b	Determined	Target not TF/ Evaluation by ESCAPE	-	-0.107	-2	1.0	1-	1-	1
Cdx2	EpiTF(Eomes,Fgf5)	Predicted (Posi/Nega)	---	-	-0.109	1	0.3	NE	1+	

b.

Source Gene	Target Gene	Actions on	Evidences (PubMed ID)	+/-	GGM	*Di,j*	*Ei,j*	LOF	GOF	ChIP
Nanog	Nanog	Curated (Self-regulation)	**15860457;**	+	0.000	2	1.4	2++		4
Klf4	Klf4	Curated (Self-regulation)	**22337869;23667633**	+	0.000	0	1.5	1+	1-	4
Tbx3	Tbx3	Curated (Self-regulation)		+	0.000	1	1.0	1+	NE	
Pou5f1	Pou5f1	Curated (Self-regulation)	**25422984;25582194**	+	0.000	2	1.7	3+++	1-	4
Cdx2	Cdx2	Curated (Self-regulation)	**18941526;**	+	0.000	1	0.3	NE	1+	
Sox2	Sox2	Curated (Self-regulation)	**16547000;17239249**	+	0.000	0	1.2	1+	1-	3
Gata6	Gata6	Curated (Self-regulation)	**11937486;18941526;**	+	0.000	NE	NE	NE	NE	NE
**Tcf3**	**Nanog**	Curated	**16894029;18347094;18483421;**	-	0.000	-2	0.6	1-	1-	2
**Nanog**	**Tcf3**	Curated	**18483421;**	+	0.000	-1	0.8	1-		3
**Pou5f1**	**Sox2**	Curated	**16153702;16518401;**	+	0.000	1	1.5	2++	1-	5
**Sox2**	**Pou5f1**	Curated	**16153702;16518401;**	+	0.000	-1	0.8		1-	4
Gata6	Nr6a1	Curated	**18941526;**	+	0.000	NE	NE	NE	NE	NE
Pou5f1	Tcf3	Curated	**18483421;**	+	0.000	-1	0.8	1-		5
Nanog	Myc	Curated	**18614019;19884255;21499299;**	-	0.000	0	0.0			
Nr6a1	Pou5f1	Curated	**11578963;11702949;**	-	0.000	NE	NE	NE	NE	NE
Tcf3	Tbx3	Curated	**18467660;**	-	0.000	-1	1.3	1-		2
Tcf3	Nr5a2	Curated	**24648413;**	-	0.000	-2	1.5	1-	1-	1
**Pou5f1**	**Cdx2**	Curated	**16153702;16518401;**	-	0.000	-2	1.1	2--		4
**Cdx2**	**Pou5f1**	Curated	**16325584;22942124**	-	0.000	-1	0.3	NE	1-	

Appendix Table S1: The regulatory relationships considered in the model and their experimental evidence.

(a) Inferred gene-to-gene relationships and (b) known regulations including self-activations which were not captured in the GRN inference. The predicted positive or negative correlation strength is indicated in the 'GGM' column. The observations from high-throughput experiments accumulated in the ESCAPE database are shown in the 'LOF/GOF/ChIP' columns. The ESCAPE-based evaluation score and positive/negative directionalities are shown in the columns indicated as '$E(i,j)$' and '$D(i,j)$', respectively. In the cases where one of the paired genes is not a TF (e.g. Smad6, Dnmt3b), the gene was assigned as a target gene of the regulatory edge. The gene pairs in bold have bi-directional regulations. The gene pairs in blue are the links whose positive/negative directions were refined by literature. The gene pairs highlighted in gray are links for which direction could not be determined in this step.

3-3. Representation of signaling pathway activities and GRNs

The activities of seven signaling pathways were considered in the PSC simulation: LIF-Stat3, BMP4-Smad1/3/5, Activin A/Nodal-Smad2/3, WNT, FGF, ERK, and PI3K. Importantly, the activity of every signaling pathway is regulated by variables in the model. For example, the expressions of genes coding autocrine proteins (e.g. *Bmp4*, *Activin A/Nodal* or *Fgf4*) are downstream targets of their own signaling pathways. Also, some signaling receptors or signaling inhibitors are predicted to be correlated with TFs or other signals: e.g. *Oct4* was predicted to be a negative regulator of both *Smad6* and *Fgfr2*, which are the components of signaling pathways under BMP4 and FGF, respectively. To model these complexities, we defined the activities of these seven signaling pathways based on the ON/OFF-values of each pathway's components, including cytokines, inhibitors or receptors as listed in Appendix Table S2. Assuming that signaling events occur faster and more deterministic than gene transcription, signaling activities were calculated at each simulation step based on the ON/OFF values of the variables, and these were used immediately in the following stochastic update for the genes under the control of signaling activities. As a consequence, we can calculate the probability of each signaling pathway being active in an SCC as a value ranging from 0 to 1 and therefore estimate the endogenous signaling activity in the associated PSC subpopulation.

Pathway	*Definition*<br>Target genes
LIF<br>(SignalLIF)	*ON or OFF (treated as input parameter)*<br>Gbx2, Klf4, Myc
WNT<br>(SignalWNT)	*(iGSK3b or SignalPI3K) and (not Cdx2) and (not Tcf3) and (not iWNT)*<br>* iGSK3b: GSK3ß inhibitor<br>Esrrb, Gbx2, Fgf4, Lefty1, Pitx2, Myc, Tcf3 (repressive effect)
BMP<br>(SignalBMP)	*Bmp4 and (not Smad6) and (not Smad7) and (not iBMP)*<br>Bmp4, EpiTFs, Smad6, Cdx2
FGF<br>(SignalFGF)	*Fgfr2 and (Fgf4 or bFGF)*<br>Fgfr2
ERK<br>(SignalERK)	*SignalFGF and (not SignalBMP) and (not iERK)*<br>Gata6, Dusp6, Myc, Tbx3 (repressive effect)
ActivinA/Nodal<br>(SignalACT)	*ActivinA-Nodal and (not Smad7) and (not iACT)*<br>ActivinA-Nodal, Nanog, Lefty1, Pitx2
PI3K<br>(SignalPI3K)	*SignalLIF and SignalFGF*<br>Tbx3

Appendix Table S2: Definition of signaling pathway activities and their downstream targets.

3-4. Consideration of epigenetic-dependent usage of Oct4 enhancer regions

There is increasing evidence of pluripotent state transitions and stabilization being driven by epigenetic mechanisms(Bao et al., 2009; ten Berge et al., 2011; Tesar et al., 2007). As a starting point to consider epigenetic effects on the GRN, we have taken into account two regulatory regions (distal enhancer and proximal promoter) of *Oct4* and the switching between them. The OCT4-SOX2 complex(Yeom et al., 1996), NANOG, KLF4, and KLF2 are known to bind to both sites and positively regulate *Oct4* expression. LRH1 was considered as a regulator for the proximal site. OCT4-CDX2 complex and GCNF bind to and repress the distal and proximal sites, respectively(Niwa, 2007). Switching between the two sites was modeled as opposing functions of *Klf2* and *Dnmt3b*. *Dnmt3b* is predominantly responsible for DNA methylation in EpiSCs(Chen et al., 2003), while *Klf2* is reported to block the methylation of the proximal promoter of *Oct4(Gillich et al., 2012)* synergistically with *Prdm14(Borgel et al., 2010)*. Under these circumstances, the switching mechanism was abbreviated and modeled as follows: the states “Dnmt3b-ON AND Klf2-OFF” use the rule for the proximal regulatory site, and the other states (“Klf2-ON OR Dnmt3b-OFF”) use the one for the distal enhancer to update Oct4 (Appendix Figure S3.1).

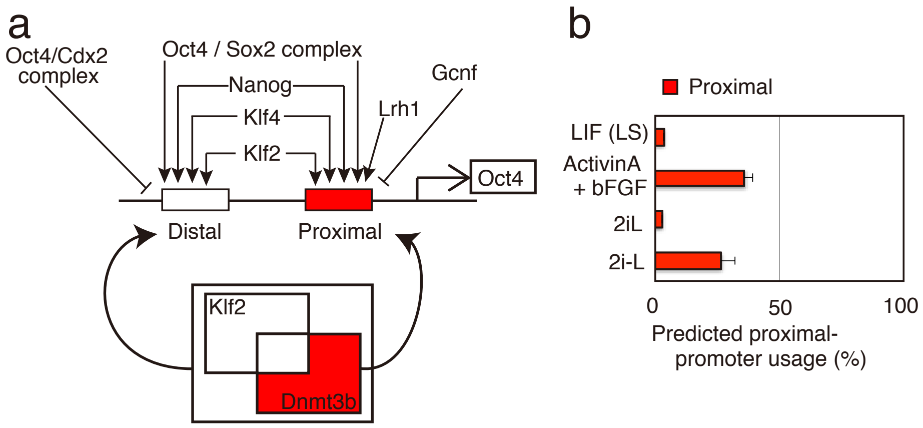

Appendix Figure S3.1. Model assumption for switching the Oct4 regulatory region between distal enhancer and proximal promoter.
a. Modeled distinct regulatory rules between Oct4 distal- and proximal-sites. **b**. Usage of the regulatory regions in the predicted pluripotent populations under distinct input conditions. In EpiSC maintenance conditions (Activin A and bFGF as inputs), proximal promoter usage is predicted to increase. In LIF+2i conditions, most of the pluripotent cells use the distal enhancer to regulate Oct4 expression.

3-5. Boolean logical functions for the regulatory edges

The regulatory edges were abstracted as Boolean logic functions such that positive and negative inputs were combined using OR-functions and AND-functions, respectively. For example, when a gene has two activators (A_1, A_2) and two repressors (R_1, R_2) as upstream regulations, the update function of a target gene will be present when one of the activators is present ($\underline{A_1\ OR\ A_2}$) while none of the repressors are present ($\underline{NOT\ R_1\ AND\ NOT\ R_2}$, or, $\underline{NOT\ (R_1\ OR\ R_2)}$). This is currently the most established rule that can widely reflect biological phenomena(Albert and Thakar, 2014; Dunn et al., 2014; Raeymaekers, 2002).

Exceptions were made for genes which code for proteins known to form a complex, such as OCT4-SOX2(Chew et al., 2005) and OCT4-CDX2(Erwin et al., 2012). As denoted in the main text, the model component 'EpiTFs' is a conceptual aggregation of the EpiSC-enriched genes, *Brachyury (T)*, *Fgf5*, *Eomes* and *Otx2*. However, some pluripotency factors have distinct effects on the EpiSC-enriched genes, resulting in a see-saw-like lineage induction(Shu et al., 2013). In the above GGM-based GRN prediction, both *Sox2* and *Klf4* have significant negative correlations with *Eomes* and *Otx2*, respectively. These negative regulatory edges from *Sox2*/*Klf4* to EpiTFs were confirmed via the ESCAPE database (Appendix Table S1). However, while experimental data from the ESCAPE database suggest that *Sox2* has a positive effect on *Otx2* expression (confirmed by two genome-binding studies, two LOF studies, and one GOF study) and *Klf4* has a positive effect on *Eomes* expression (confirmed by two genome-binding studies and one LOF study), the overall negative regulation of the aggregate EpiTFs by both *Sox2* and *Klf4* outweigh these individual discrepancies. We therefore modeled (Sox2 AND Klf4) as a repressor of EpiTFs. It was also inferred that both *Sox2*

and *Klf4* are required to repress the aggregated EpiTF activity. We therefore modeled *(Sox2 AND Klf4)* as a repressor of EpiTFs.

In the model, LIF signaling activity was modeled as an input parameter, can only take on ON or OFF values. However, the strength of the signaling effect may differ among its direct downstream targets (*Klf4*, *Gbx2* and *Myc*). Given this and to expand the space of the model predictions, we considered three types of Boolean functions for these genes, which were optimized in the following section 3-6:

F1: *Signal AND* $(A_1$ *OR* $A_2)$	Both *Signal* and one activator required
F2: *Signal OR* A_1 *OR* A_2	Either *Signal* or one activator required
F3: *Signal OR* $(A_1$ *AND* $A_2)$	Either *Signal* or both activators required

The signal has greater impact on the gene state for functions F1 and F3 than for F2. The presence of the signal is necessary in F1, but is sufficient in F2 and F3 (Appendix Figure S.3.2).

Signal	ON	ON	ON	ON	OFF	OFF	OFF	OFF
A1	ON	ON	OFF	OFF	ON	ON	OFF	OFF
A2	ON	OFF	ON	OFF	ON	OFF	ON	OFF

F1	ON	ON	ON	OFF	OFF	OFF	OFF	OFF
F2	ON	ON	ON	ON	ON	ON	ON	OFF
F3	ON	ON	ON	ON	ON	OFF	OFF	OFF

Appendix Figure S.3.2. Boolean Truth table for the functions F1-F3.
Current profile of signal and two activators (upper) and the resultant gene state in the following Boolean update (lower).

3-6. Consensus Gene Regulatory Networks in mESCs

Based on the steps described above, we confirmed most of the regulatory edges and assigned Boolean logic functions to them. The remaining uncertainties (10 edge directionalities and three Boolean function options on three genes in section 3-5) gave us 27,648 possible models. To define one consensus model among the possibilities, we took a best-fit strategy where the simulated gene expression levels were compared with experimentally-measured single cell gene expression frequencies. We employed published single cell RNA-seq data(Kolodziejczyk et al., 2015) and qPCR data(MacArthur et al., 2012) on mESCs in LIF+Serum (LS) for model evaluation. mRNA expression (either fpkm, Fragments Per Kilobase of transcript per Million mapped reads for RNA-seq; or ddCt, delta-delta Ct value for qPCR) in single cells were binarized into ON (gene expressing) and OFF (gene non-expressing) clusters by applying k-means clustering on the expression data across all samples with k=2. The frequency of gene-expressing single-cells in the population was then calculated. As there are two series of single-cell transcriptomic profiles in the reference, we took the average levels of the two datasets. The expression level value for EpiTFs was calculated by taking mean levels of *Otx2*, *Eomes* and *Fgf5*.

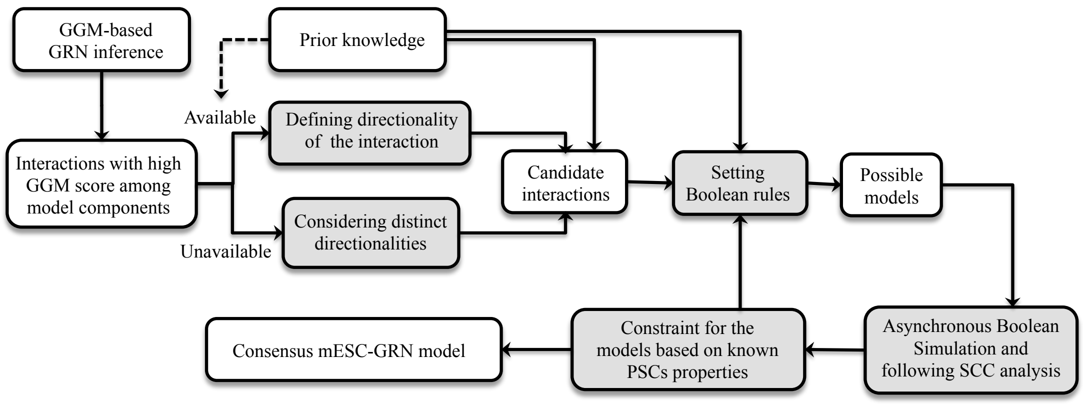

Appendix Figure S3.3. A diagram to define consensus mESC-GRN.
Gray rectangles indicate the operative processes, while white rectangle indicate key objects to define consensus mESC-GRN and its model.

All the candidate models were simulated for the LIF/Serum (LS) condition, and the best-fit model was selected to minimize the total Euclidean distance between simulation and experimental data for all genes (Appendix Figure S3.3 and Figure S2a). The full representation of this best-fit model model is in Supplementary Table S1.

4. Characterization of PSCs via pluripotency, sustainability, and susceptibility

Pluripotency is supported in part by the expression of core transcription factor network components *Oct4*, *Sox2*, and *Nanog*. These three core TFs (OSN) are positively regulated by other supporting TFs, *Esrrb*, *Klf2/4*, and *Tbx3*. Alternatively, spontaneous expression of lineage-specific TFs—*Cdx2* and *Gata6*—and early differentiation markers—*Brachyury (T)*, *Fgf5*, *Eomes*, and *Otx2*—is also observed in mESCs. These components are known to have bidirectional inhibitory relationships with *Oct4*, *Nanog*, and *Sox2*. This competition between TFs gives rise to fluctuations in gene expression over time. PSC populations with higher, more homogeneous OSN expression overcome these fluctuations and are surmised to have more robust characteristics of pluripotency. We measured this strength of pluripotency using a *pluripotency* score that is a summation of OSN expression levels averaged across the whole population: $p_{Oct4} + p_{Sox2} + p_{Nanog}$.

The *sustainability* score can be used to estimate the stability of PSC populations, defined as a single, large SCC in a given input condition. *Sustainability* reflects how intrinsically stable the GRN is over time in the absence of any extrinsic perturbations. The *sustainability* score is defined as a subtraction of this out-going cell probability from 1, thus it also ranges from 0 to 1 (see Section 1-4). We applied the thresholds for SCCs to be considered in the analysis as those which demonstrate a *sustainability* score > 0.7. This was based on the assumption that a more stable PSC is more likely to persist over time and that it will become a larger determinant of population-average expression levels.

Alternatively, if the PSC-GRN stabilized under a given input condition is structurally sensitive to extrinsic stimuli, such as perturbations in either signaling pathway activities or gene expression levels, the PSCs will alter gene expression profiles and change into a different state. This

concept of susceptibility to extrinsic stimuli was quantified using a *susceptibility* score. High *susceptibility* of the *pluripotency* score may reflect a higher probability of exiting pluripotency (i.e. differentiation commitment or cell death). It follows that reduced susceptibility to a specific stimulus (or set of stimuli) indicates robustness of the GRN towards the stimulus, due in part to support by network redundancy or feedback motifs. *Susceptibility* is measured as the change of variance of the gene expression profiles in PSCs after removing individual regulatory edges from the model:.

$$susceptiblityscore = \frac{1}{N} \cdot \sum_{i}^{N} \sqrt{\left(\frac{p_i' - p_{i,cont}}{p_{i,cont}}\right)^2}$$

where p_i' is a level of the gene after removal of the edge i and $p_{i,cont}$ is that of un-perturbed model, and *N* denotes number of regulatory edges. To assess susceptibility towards input signal conditions, the coefficient of variation (CV) for the genes of interest is calculated for each of the perturbed models.

Metrics	Definitions
**Pluripotency score**	PSC population with higher (homogeneous) OSN expression has more robust characteristics of pluripotency and higher differentiation potential to multiple lineages.
**Sustainability**	PSC population where single cells (profiles) are more likely to remain within the population over time (i.e. less likely to escape the metastable state) has higher sustainability.
**Susceptibility**	If redundant regulatory interactions confer robustness to the PSC state (i.e. gene expression pattern), the population is less sensitive to extrinsic perturbations.<br>Meanwhile, a PSC population with high susceptibility is more sensitive to perturbations in signal activity or genetic manipulations, and thus has a higher chance of differentiating upon small perturbations.

Appendix Table S3. PredicAppendix Table Setrics of PSCs and their biological relevancies.

5. Simulation of stabilized PSC populations in various culture conditions

5-1. *In silico* and *in vitro* manipulation of signal inhibition

To predict the impact of manipulating exogenous signaling using inhibitory small molecules, we defined inhibitory (i)-Signals for ERK, Activin A/Nodal, BMP4, and WNT signaling. Signaling activity is defined as follows: *Signal activity = (Signal components) and (NOT i-Signal)*. As small molecule-based inhibition cannot completely eliminate basal signaling activity experimentally, we set i-Signal as a random value whose status is determined randomly and independently from the other

network components. It follows that the average probability of i-Signal in each SCC is around 0.5. In this way, the signaling activity *in silico* decreases but does not vanish. The default settings of i-Signals are OFF, except in the cases where inhibitors are specifically added.

Due to the switch-like response observed in LIF signaling, we did not include an i-Signal and defined only ON (1) or OFF (0) inputs. As a result the *in silico* LIF-OFF condition corresponds both to removal of exogenous LIF and to active suppression of LIF signaling using an inhibitor to Janus Kinase (JAKi). The signal manipulation settings we explored are listed in Appendix Table S4.

Note that the JAK inhibitor utilized in this study has been used in a number of studies as a blocker of endogenous LIF/Stat3 in mESCs(Lo Nigro et al., 2017; Yi and Merrill, 2010) and other cell types(Huen et al., 2015). The concentration of the JAK inhibitor in those studies is 1 uM or lower, and showed sufficient and saturated inhibitory effects on Stat3 phosphorylation in doses higher than 1 uM. We set the concentration of the JAK inhibitor as high as 2uM and showed the clear difference between 2i+Jaki+Bmp4+Alki and 2i+Bmp4+Alki in their OCT4/CDX2 expression profiles, which indicates its efficient inhibitory effect on the LIF/Stat3 pathway.

Signaling pathway	Effect	Symbol	Experimental manipulation	*In silico* manipulation
LIF-pStat3	Activation	+L	LIF	LIF = ON (1)
LIF-pStat3	Inhibition	–L	JAKi	LIF = OFF (0)
Wnt-canonical	Activation	+W	CH	iGSK3b = ON(1)
Wnt-canonical	Inhibition	–W	Dkk1	Define (not iWnt), iWnt = Random
BMP4-pSmad1	Activation	+B	BMP4	Bmp4 = ON (1)
BMP4-pSmad1	Inhibition	–B	LDN	Define (not iBmp), iBmp = Random
ActivinA/Nodal	Activation	+W	ActivinA	Activin = ON (1)
ActivinA/Nodal	Inhibition	–W	Alki	Define (not iActivin), iActivin = Random
FGF	Activation	+E	bFGF	bFGF = ON (1)
ERK	Inhibition	–E	PD	Define (not iERK), iERK = Random

Appendix Table S4: Experimental and *in silico* manipulation of signals using cytokines and small molecules.

5-2. Simulation for the input conditions that mimic known PSC populations

We simulated four conditions where PSCs can be stably maintained: 1) mESCs in LIF+Serum (LS), 2) naïve mESCs in LIF+2i (2iL), 3) naïve mESCs in 2i without LIF (2i-L), and 4) EpiSCs in bFGF+Activin (bF+A). Note that as Activin A and BMP4 (and Fgf4) are secreted in an autocrine manner, they are considered to be variable (i.e. set as random) when no cytokines are added to the medium. These control conditions are defined as shown in Appendix Table S5.

Conditions	Experiments	Simulation inputs	
		ON	OFF
LS	LIF	LIF	bFGF
			iGSK3b
			iERK
2iL	LIF	LIF	bFGF
	CH	iGSK3b	
	PD	iERK	
2i-L / 2iJ	Jaki	iGSK3b	LIF
	CH	iERK	bFGF
	PD		
bF+A	Activin A	ActivinA/Nodal	LIF
	bFGF	bFGF	CH
			iERK

Appendix Table S5: Settings of control PSC conditions in the model and experiments.

5-3. Robustness of calculated population-average gene expression levels

For this study, we performed R-ABS from 700 randomly set initial profiles with 700 updates from each profile. The high reproducibility of the simulation results initiated with a lower number of input profiles (300 profiles, Appendix Figure S5) demonstrates the robustness of the simulation outcome. Moreover, the predictions of average gene expression levels were robust against varying thresholds for SCC classification. When changing the minimum SCC size threshold from 1 to 30 (default = 10), the differences in predicted Oct4 level were as small as ±0.0%, ±0.0%, ±1.58% and ±1.69, for LS, 2iL, 2i-L and EpiSC conditions, respectively. when changing the minimum sustainability threshold from 0.0 to 0.7 (default = 0.7), the differences in predicted Oct4 level were ±0.0%, ±0.0%, ±0.16% and ±0.77% for the same conditions. We anticipate that the robustness reflects the high reachability of random initial profiles to the SCC, which is analogous to the population-level stabilization of PSCs by culture conditions *in vitro*.

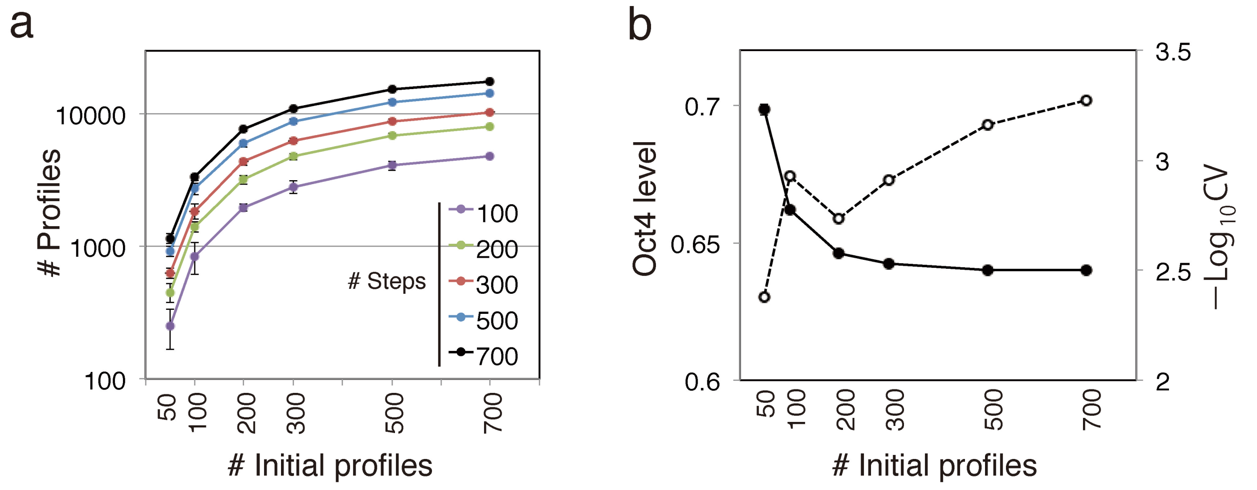

Appendix Figure S5-1. Representative calculation robustness of population-average gene expression profile using control LS condition as an example.
a. Changes in the size of SCC (# profiles) depending on the number of steps (i.e. updates) in one simulation run with varied number of initial profiles. Sustainability scores are 1.0 for all simulation settings. The results of five independent simulations under the condition of LS (LIF+serum) are shown. **b.** Change of variance (the second axis, CV: Standard deviations divided by the mean value) of calculated expression level of Oct4 (first axis) averaged from five independent simulations where the number of steps (updates) in each simulation was set to 700.

5-4. Validity of the reconstructed model

We compared the model's predictions of OSN levels in the same signal conditions as those in Fig. 4f, both with and without 10 edges not validated in the literature or ESCAPE (highlighted in gray in Appendix Table S1). As shown in Appendix Figure S5-2, the partial model, which is the model excluding the above 10 interactions, failed to predict the expression levels of *Sox2* and *Nanog* in the LS (orange triangle), LIF+Dkk+PD (gray circles) and LIF+2i conditions (red circles and triangle) measured by immuno-fluorescent microscopy. The fitting scores based on R-square values were 0.63 (*Oct4*), 0.76 (*Sox2*) and 0.71 (*Nanog*) for the full model (Fig. 4f), while the scores for the partial model were 0.38 (*Oct4*), 0.03 (*Sox2*) and 0.26 (*Nanog*). This result strongly supports the validity of the 10 novel edges in our PSC model which were predicted without supporting literature or ESCAPE evidence.

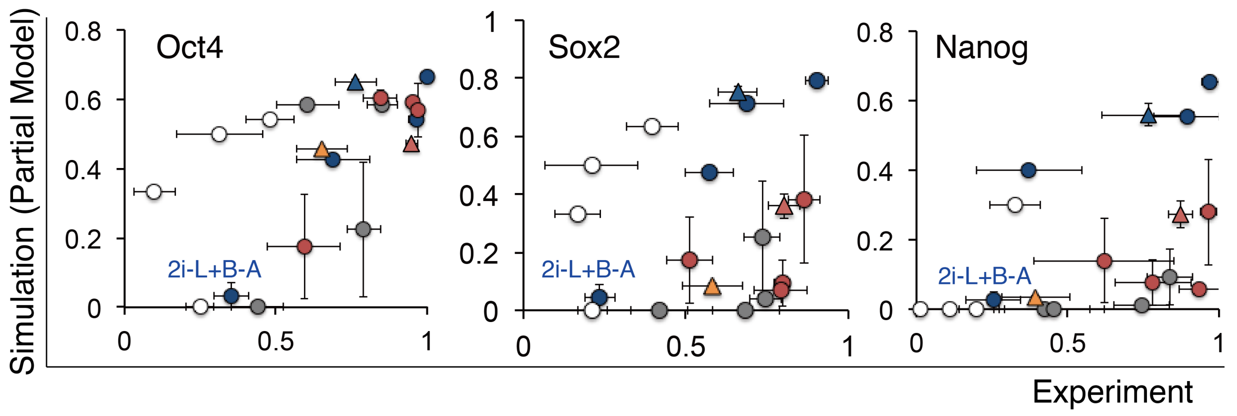

Appendix Figure S5-2. Expression frequency of OSN.

Observed (x-axis) and predicted using the partial model without predicted edges (y-axis). Each condition is tested under activated and repressed Activin A/Nodal- and BMP-signals by addition of cytokines or inhibitors (±A±B).
Circles are 16 combinatorial signal conditions indicated in Fig. 4f and triangles are the three control PSC conditions, 2iL(red), 2i-L(blue), and LS(green), respectively.

In addition to the effects of the novel edges, we tested whether manual curation of genes was necessary for our experimentally verified predictions. We “deleted” single or double genes *in silico* from the full model and directly compared the simulated results to experimental data. As shown in Appendix Figure S5-3, the genes we tested (*Esrrb*, *Gbx2*, *Klf2*, *Jarid2*, *Lefty1*, *Mycn* and *Pitx2*) and their combinations were not generally necessary to predict the *Oct4* expression profile under control PSC conditions including LS, 2iL, 2i-L and bF+A. One exception to this observation was that our *Jarid2*-null model did not yield the experimentally verified EpiSC-related (Oct4 positive) SCC in the simulated bF+A condition. Importantly, all models with combinatorial deletions failed to predict the upregulation of *Cdx2* in the 2i-L+B-A condition, even though this response was robustly validated using five independent experimental strategies (single cell flow, qPCR, RNAseq, reporter line measurements and immunohistochemistry).

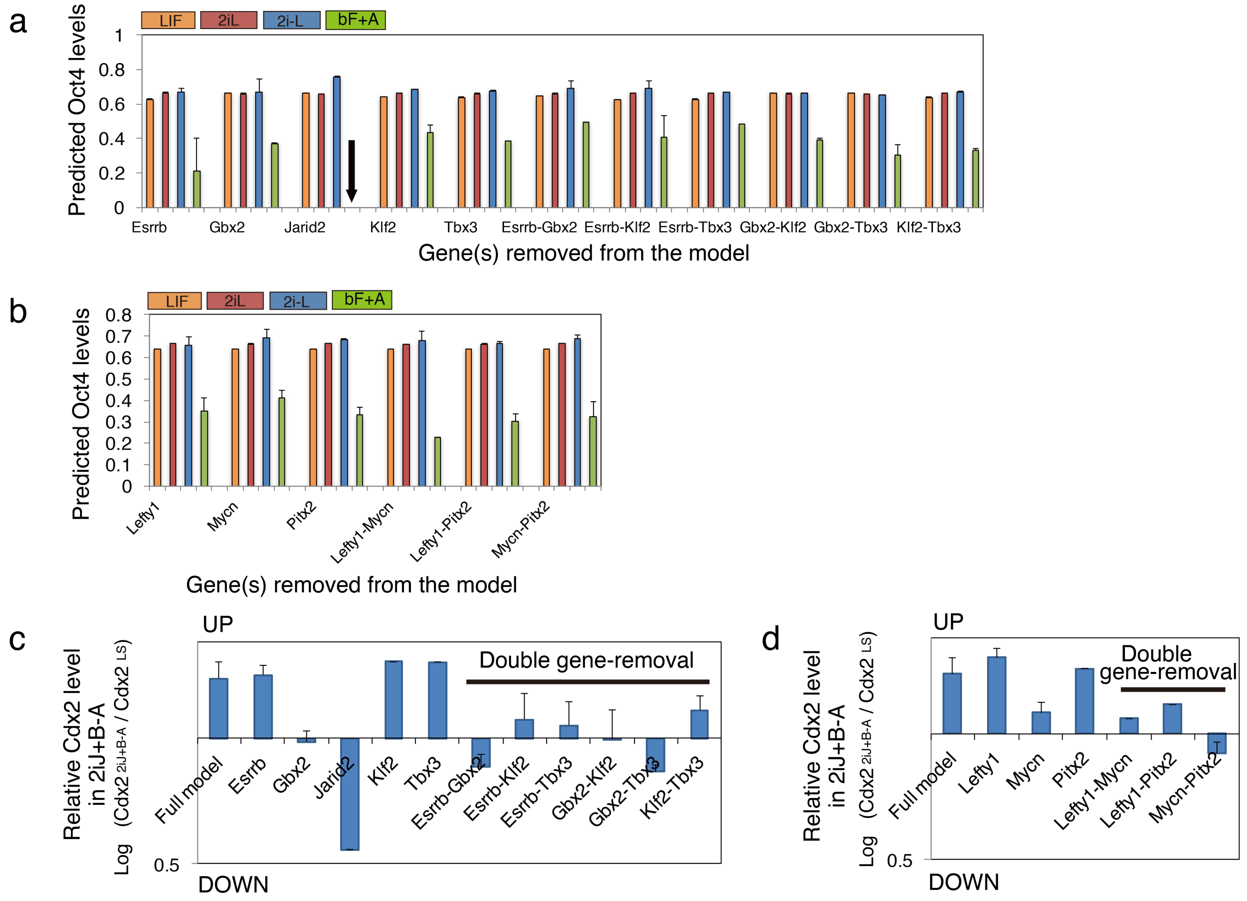

Appendix Figure S5-3. Effects of gene deletion from the original (full) model.
(a) and (b): Predicted Oct4 levels in control PSC conditions (LS; orange, 2iL;red , 2i-L; blue and EpiSC (bFGF+Activin A); green). (c) and (d): Predicted change in Cdx2 levels between LS and 2i-L+B-A condition

References

Albert, R., and Thakar, J. (2014). Boolean modeling: a logic-based dynamic approach for understanding signaling and regulatory networks and for making useful predictions. Wiley Interdiscip. Rev. Syst. Biol. Med. *6*, 353–369.

Bao, S., Tang, F., Li, X., Hayashi, K., Gillich, A., Lao, K., and Surani, M.A. (2009). Epigenetic reversion of post-implantation epiblast to pluripotent embryonic stem cells. Nature *461*, 1292–1295.

ten Berge, D., Kurek, D., Blauwkamp, T., Koole, W., Maas, A., Eroglu, E., Siu, R.K., and Nusse, R. (2011). Embryonic stem cells require Wnt proteins to prevent differentiation to epiblast stem cells. Nat. Cell Biol. *13*, 1070–1075.

Borgel, J., Guibert, S., Li, Y., Chiba, H., Schübeler, D., Sasaki, H., Forné, T., and Weber, M. (2010). Targets and dynamics of promoter DNA methylation during early mouse development. Nat. Genet. *42*, 1093–1100.

Chen, T., Ueda, Y., Dodge, J.E., Wang, Z., and Li, E. (2003). Establishment and maintenance of genomic methylation patterns in mouse embryonic stem cells by Dnmt3a and Dnmt3b. Mol. Cell. Biol. *23*, 5594–5605.

Chew, J.-L., Loh, Y.-H., Zhang, W., Chen, X., Tam, W.-L., Yeap, L.-S., Li, P., Ang, Y.-S., Lim, B., Robson, P., et al. (2005). Reciprocal transcriptional regulation of Pou5f1 and Sox2 via the Oct4/Sox2 complex in embryonic stem cells. Mol. Cell. Biol. *25*, 6031–6046.

Chickarmane, V., and Peterson, C. (2008). A computational model for understanding stem cell, trophectoderm and endoderm lineage determination. PloS One *3*, e3478.

De Cegli, R., Iacobacci, S., Flore, G., Gambardella, G., Mao, L., Cutillo, L., Lauria, M., Klose, J., Illingworth, E., Banfi, S., et al. (2013). Reverse engineering a mouse embryonic stem cell-specific transcriptional network reveals a new modulator of neuronal differentiation. Nucleic Acids Res. *41*, 711–726.

De Los Angeles, A., Ferrari, F., Xi, R., Fujiwara, Y., Benvenisty, N., Deng, H., Hochedlinger, K., Jaenisch, R., Lee, S., Leitch, H.G., et al. (2015). Hallmarks of pluripotency. Nature *525*, 469–478.

Dunn, S.-J., Martello, G., Yordanov, B., Emmott, S., and Smith, A.G. (2014). Defining an essential transcription factor program for naïve pluripotency. Science *344*, 1156–1160.

Erwin, J.A., del Rosario, B., Payer, B., and Lee, J.T. (2012). An ex vivo model for imprinting: mutually exclusive binding of Cdx2 and Oct4 as a switch for imprinted and random X-inactivation. Genetics *192*, 857–868.

Faith, J.J., Hayete, B., Thaden, J.T., Mogno, I., Wierzbowski, J., Cottarel, G., Kasif, S., Collins, J.J., and Gardner, T.S. (2007). Large-scale mapping and validation of Escherichia coli transcriptional regulation from a compendium of expression profiles. PLoS Biol. *5*, e8.

Filipczyk, A., Marr, C., Hastreiter, S., Feigelman, J., Schwarzfischer, M., Hoppe, P.S., Loeffler, D., Kokkaliaris, K.D., Endele, M., Schauberger, B., et al. (2015). Network plasticity of pluripotency transcription factors in embryonic stem cells. Nat. Cell Biol. *17*, 1235–1246.

Gillich, A., Bao, S., Grabole, N., Hayashi, K., Trotter, M.W.B., Pasque, V., Magnúsdóttir, E., and Surani, M.A. (2012). Epiblast stem cell-based system reveals reprogramming synergy of germline factors. Cell Stem Cell *10*, 425–439.

Gu, P., Goodwin, B., Chung, A.C.-K., Xu, X., Wheeler, D.A., Price, R.R., Galardi, C., Peng, L., Latour, A.M., Koller, B.H., et al. (2005). Orphan nuclear receptor LRH-1 is required to maintain Oct4 expression at the epiblast stage of embryonic development. Mol. Cell. Biol. *25*, 3492–3505.

Guo, G., and Smith, A. (2010). A genome-wide screen in EpiSCs identifies Nr5a nuclear receptors as potent inducers of ground state pluripotency. Dev. Camb. Engl. *137*, 3185–3192.

Hase, T., Ghosh, S., Yamanaka, R., and Kitano, H. (2013). Harnessing diversity towards the reconstructing of large scale gene regulatory networks. PLoS Comput. Biol. *9*, e1003361.

Hata, A., Lagna, G., Massagué, J., and Hemmati-Brivanlou, A. (1998). Smad6 inhibits BMP/Smad1 signaling by specifically competing with the Smad4 tumor suppressor. Genes Dev. *12*, 186–197.

Heng, J.-C.D., Feng, B., Han, J., Jiang, J., Kraus, P., Ng, J.-H., Orlov, Y.L., Huss, M., Yang, L., Lufkin, T., et al. (2010). The nuclear receptor Nr5a2 can replace Oct4 in the reprogramming of murine somatic cells to pluripotent cells. Cell Stem Cell *6*, 167–174.

Huen, S.C., Huynh, L., Marlier, A., Lee, Y., Moeckel, G.W., and Cantley, L.G. (2015). GM-CSF Promotes Macrophage Alternative Activation after Renal Ischemia/Reperfusion Injury. J. Am. Soc. Nephrol. JASN *26*, 1334–1345.

Kolodziejczyk, A.A., Kim, J.K., Tsang, J.C.H., Ilicic, T., Henriksson, J., Natarajan, K.N., Tuck,

A.C., Gao, X., Bühler, M., Liu, P., et al. (2015). Single Cell RNA-Sequencing of Pluripotent States Unlocks Modular Transcriptional Variation. Cell Stem Cell *17*, 471–485.

Lee, K.L., Lim, S.K., Orlov, Y.L., Yit, L.Y., Yang, H., Ang, L.T., Poellinger, L., and Lim, B. (2011). Graded Nodal/Activin Signaling Titrates Conversion of Quantitative Phospho-Smad2 Levels into Qualitative Embryonic Stem Cell Fate Decisions. PLOS Genet *7*, e1002130.

Lewis, S.L., Khoo, P.-L., De Young, R.A., Steiner, K., Wilcock, C., Mukhopadhyay, M., Westphal, H., Jamieson, R.V., Robb, L., and Tam, P.P.L. (2008). Dkk1 and Wnt3 interact to control head morphogenesis in the mouse. Dev. Camb. Engl. *135*, 1791–1801.

Liu, X., Zhang, X., Zhan, Q., Brock, M.V., Herman, J.G., and Guo, M. (2012). CDX2 serves as a Wnt signaling inhibitor and is frequently methylated in lung cancer. Cancer Biol. Ther. *13*, 1152–1157.

Lo Nigro, A., de Jaime-Soguero, A., Khoueiry, R., Cho, D.S., Ferlazzo, G.M., Perini, I., Abon Escalona, V., Aranguren, X.L., Chuva de Sousa Lopes, S.M., Koh, K.P., et al. (2017). PDGFRα(+) Cells in Embryonic Stem Cell Cultures Represent the In Vitro Equivalent of the Pre-implantation Primitive Endoderm Precursors. Stem Cell Rep.

Loh, Y.-H., Wu, Q., Chew, J.-L., Vega, V.B., Zhang, W., Chen, X., Bourque, G., George, J., Leong, B., Liu, J., et al. (2006). The Oct4 and Nanog transcription network regulates pluripotency in mouse embryonic stem cells. Nat. Genet. *38*, 431–440.

Ma, S., Gong, Q., and Bohnert, H.J. (2007). An Arabidopsis gene network based on the graphical Gaussian model. Genome Res. *17*, 1614–1625.

MacArthur, B.D., Sevilla, A., Lenz, M., Müller, F.-J., Schuldt, B.M., Schuppert, A.A., Ridden, S.J., Stumpf, P.S., Fidalgo, M., Ma'ayan, A., et al. (2012). Nanog-dependent feedback loops regulate murine embryonic stem cell heterogeneity. Nat. Cell Biol. *14*, 1139–1147.

Marbach, D., Costello, J.C., Küffner, R., Vega, N.M., Prill, R.J., Camacho, D.M., Allison, K.R., The DREAM5 Consortium, Kellis, M., Collins, J.J., et al. (2012). Wisdom of crowds for robust gene network inference. Nat. Methods *9*, 796–804.

Margolin, A.A., Nemenman, I., Basso, K., Wiggins, C., Stolovitzky, G., Dalla Favera, R., and Califano, A. (2006a). ARACNE: an algorithm for the reconstruction of gene regulatory networks in a mammalian cellular context. BMC Bioinformatics *7 Suppl 1*, S7.

Margolin, A.A., Wang, K., Lim, W.K., Kustagi, M., Nemenman, I., and Califano, A. (2006b). Reverse engineering cellular networks. Nat. Protoc. *1*, 662–671.

Masui, S., Ohtsuka, S., Yagi, R., Takahashi, K., Ko, M.S.H., and Niwa, H. (2008). Rex1/Zfp42 is dispensable for pluripotency in mouse ES cells. BMC Dev. Biol. *8*, 45.

Niwa, H. (2007). How is pluripotency determined and maintained? Dev. Camb. Engl. *134*, 635–646.

Qi, J., and Michoel, T. (2012). Context-specific transcriptional regulatory network inference from global gene expression maps using double two-way t-tests. Bioinforma. Oxf. Engl. *28*, 2325–2332.

Raeymaekers, L. (2002). Dynamics of Boolean networks controlled by biologically meaningful functions. J. Theor. Biol. *218*, 331–341.

Shu, J., Wu, C., Wu, Y., Li, Z., Shao, S., Zhao, W., Tang, X., Yang, H., Shen, L., Zuo, X., et al. (2013). Induction of pluripotency in mouse somatic cells with lineage specifiers. Cell *153*, 963–975.

Singer, Z.S., Yong, J., Tischler, J., Hackett, J.A., Altinok, A., Surani, M.A., Cai, L., and Elowitz, M.B. (2014). Dynamic heterogeneity and DNA methylation in embryonic stem cells. Mol. Cell *55*, 319–331.

Takahashi, K., and Yamanaka, S. (2006). Induction of pluripotent stem cells from mouse embryonic and adult fibroblast cultures by defined factors. Cell *126*, 663–676.

Tam, W.-L., Lim, C.Y., Han, J., Zhang, J., Ang, Y.-S., Ng, H.-H., Yang, H., and Lim, B. (2008). T-cell factor 3 regulates embryonic stem cell pluripotency and self-renewal by the transcriptional control of multiple lineage pathways. Stem Cells Dayt. Ohio *26*, 2019–2031.

Tesar, P.J., Chenoweth, J.G., Brook, F.A., Davies, T.J., Evans, E.P., Mack, D.L., Gardner, R.L., and McKay, R.D.G. (2007). New cell lines from mouse epiblast share defining features with human embryonic stem cells. Nature *448*, 196–199.

Thisse, C., and Thisse, B. (1999). Antivin, a novel and divergent member of the TGFbeta superfamily, negatively regulates mesoderm induction. Dev. Camb. Engl. *126*, 229–240.

Varlakhanova, N.V., Cotterman, R.F., deVries, W.N., Morgan, J., Donahue, L.R., Murray, S.,

Knowles, B.B., and Knoepfler, P.S. (2010). myc maintains embryonic stem cell pluripotency and self-renewal. Differ. Res. Biol. Divers. *80*, 9–19.

Yan, X., Liu, Z., and Chen, Y. (2009). Regulation of TGF-ß signaling by Smad7. Acta Biochim. Biophys. Sin. *41*, 263–272.

Yeom, Y.I., Fuhrmann, G., Ovitt, C.E., Brehm, A., Ohbo, K., Gross, M., Hübner, K., and Schöler, H.R. (1996). Germline regulatory element of Oct-4 specific for the totipotent cycle of embryonal cells. Dev. Camb. Engl. *122*, 881–894.

Yi, F., and Merrill, B.J. (2010). Non-cell-autonomous stimulation of stem cell proliferation following ablation of Tcf3. Exp. Cell Res. *316*, 1050–1060.
