# Supplementary material for: Modeling signaling‐dependent pluripotency with Boolean logic to predict cell fate transitions
Source: Mol Syst Biol. 2018 Jan 29;14(1):e7952. doi: 10.15252/msb.20177952 (PMC5787708; doi:10.15252/msb.20177952)
Supplement: Supplementary file 2 — Expanded View Figures PDF [file MSB-14-e7952-s002.pdf]

Expanded View Figures

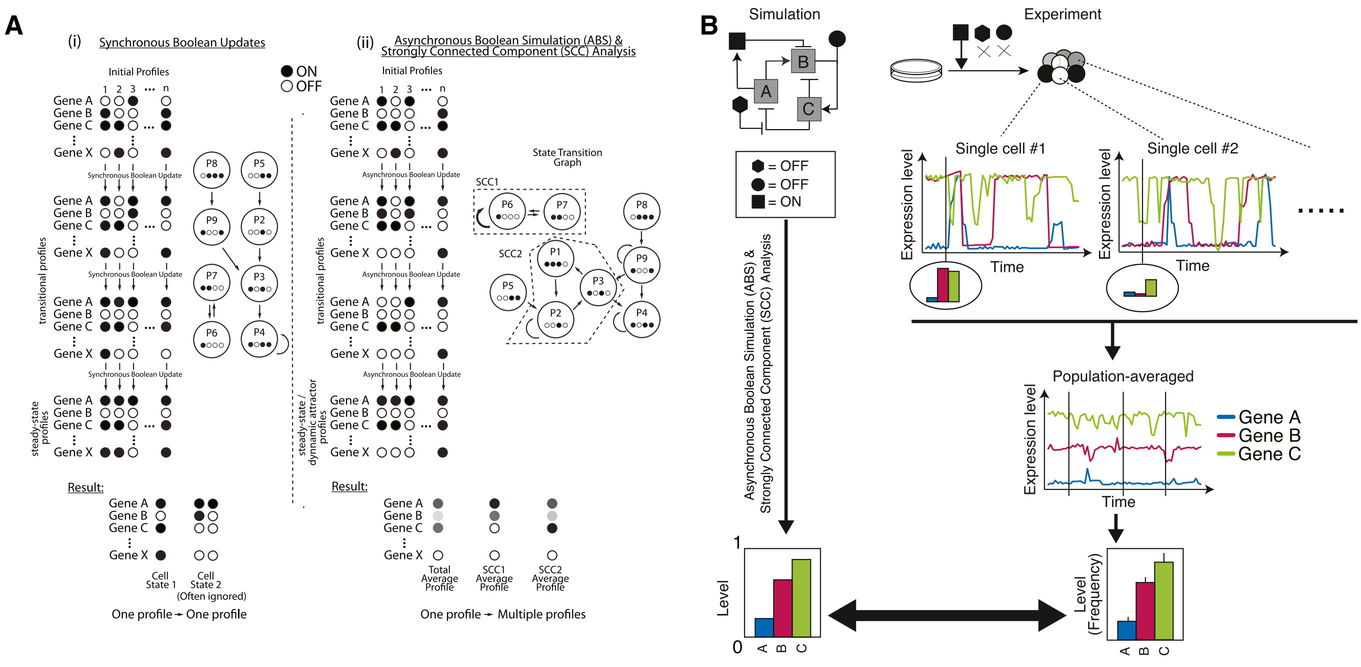

Figure EV1. General strategy of the simulation.

A The properties of synchronous (i) and asynchronous (ii) Boolean simulation. Importantly in ABS, a single starting profile can give rise to multiple different resultant profiles and therefore all transitional profiles are represented with probabilities. Note that we used random ABS where the genes updated in each transition are set randomly to reduce the calculation cost.

B The background of our simulation framework: heterogeneity derived from single-cell fluctuations and stabilization of PSC populations showing robust gene expression pattern under a certain signaling input condition (depicted with black geometric shapes).

© 2018 The Authors

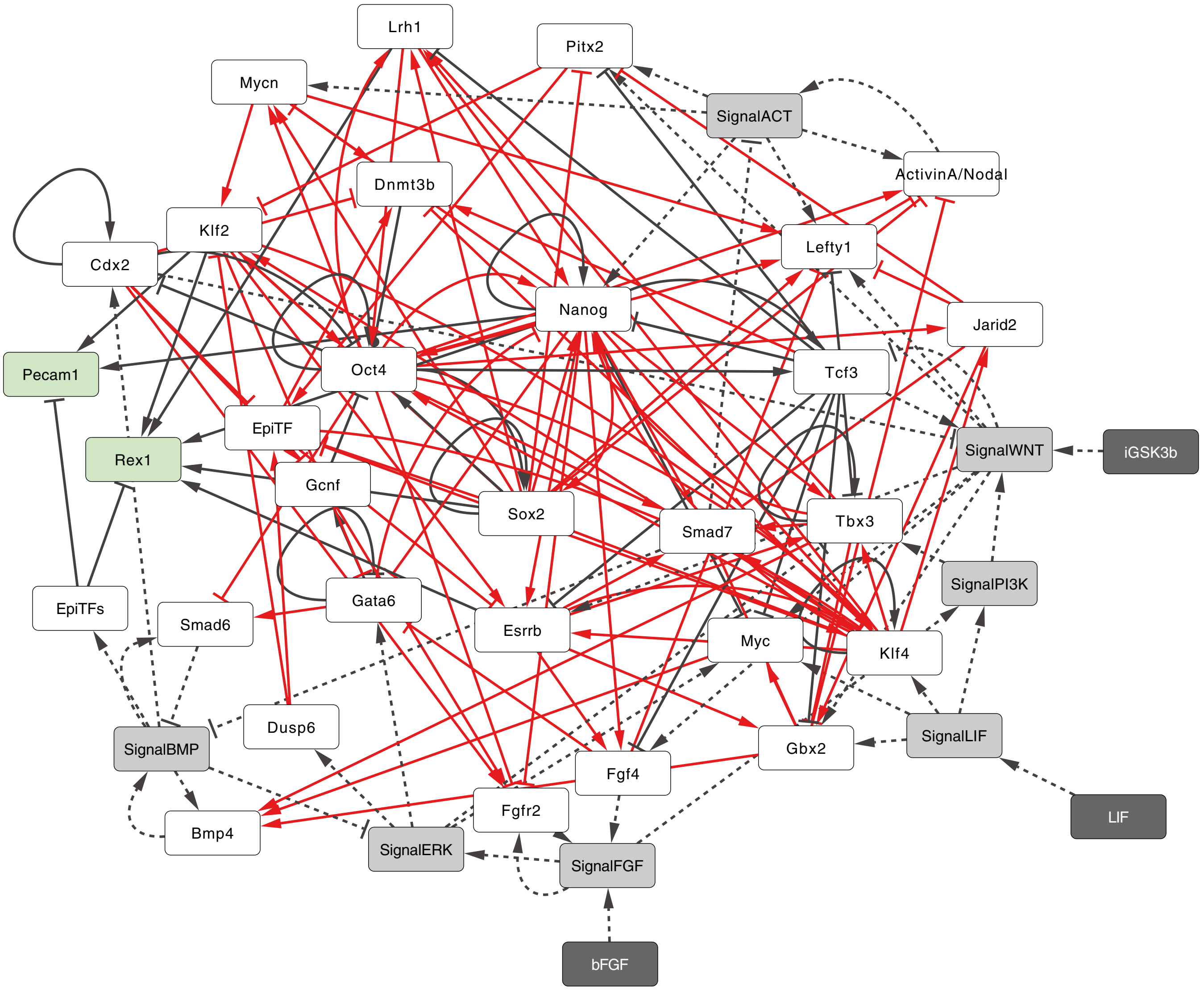

Figure EV2. Modeling of mESC-GRN; related to Fig 2.

A network view of the mESC-GRN model where rectangles indicate model components including genes (white and green white and green for genes with and without outgoing regulatory interactions), signaling activities (gray), and cytokines or small molecules as inputs to control signaling activities (dark gray). Edges between rectangles represent the regulatory relationships between genes (solid lines) and within signaling pathways (dotted lines). The edge color indicates either literature curation-based (black) or inferred/predicted (red) regulations.

© 2018 The Authors

Figure EV3. Comparison of predicted and experimentally observed data on gene expression patterns in distinct PSCs; related to Fig 3.

A Predicted population-averaged expression level (mean of five independent simulations) for each pluripotency-associated gene in the control LS condition is comparable to the frequency of gene-expressing cells from the reported single-cell measurements using RNA-seq (triangle; Kolodziejczyk *et al*, 2015) and fluidigm-qPCR (circle; MacArthur *et al*, 2012). The gene expression levels were binarized into ON or OFF for each single cell to calculate frequencies. The error bars for the simulation resutls represent s.d. of five independent simulations, whereas those for the single-cell RNA-seq data represent s.d. of two replicates.

B Comparison of predicted co-expressions of epiblast-specific genes (EpiTFs—Eomes, Otx2, and Fgf5) with O/S/N and those measured by qPCR-based single-cell mRNA data. Oct4 and Nanog are likely to be co-expressed with EpiTFs, whereas Sox2 is not, as it shows a negative correlation with EpiTFs (i.e., EpiTF expression in Sox2— cells is higher) in both our simulation and in published, single-cell expression data (Hayashi *et al*, 2008; MacArthur *et al*, 2012). *P*-value was calculated by Fisher's exact test. The error bars represent s.d. of five independent simulations.

C Simulation of distinct PSCs recapitulates population-level gene expression measurements. All expression data are scaled relative to respective gene expression in control LS conditions. The hierarchical clustering with AU (approximately unbiased) *P*-value and BP (bootstrap probability) value based on multiscale bootstrap resampling was carried out with the pvclust package in R. The results are displayed in the pinwheel view in Fig 3C, where upper and lower limits are set to +5 and −10, respectively, in the simulation to avoid singularities caused by null expression. The experimental data were taken from indicated GEO entries, and RNA-seq data for EpiSCs were kindly provided by Dr. Ronald Mckay. Cell lines used are 129 (GSE15603 and GSE62155), J1 (GSE58735), ESF175/1, ESF58/2, ESF122 (GSE7902) cell lines, or mESCs were derived from C57BL/6J strain blastocysts (GSE53275).

D *In silico* GOF/LOF study in EpiSC (bF+A) or mESC (LS) conditions. All SCCs above ten profiles and sustainability > 0.7 are shown, and the gene expression levels of each component in each SCC are color-coded between blue (0.0) to yellow (1.0). The population-averaged expression levels based on the GOF/LOF results were shown in the PCA metrics in Fig 3D.

E Predictions (left) and measurements (right) of population-averaged expression levels of OSN in EpiSC conditions (bF+A) increased in response to extrinsic manipulation of BMP4. BMP4 was set as continuous-ON (EpiSC + BMP4) and random (EpiSC). The frequencies reported for Oct4, Sox2, and Nanog-positive cells, assessed by Cellomics high content screening, represent the mean and s.d. of four replicates. Asterisk indicates the significant difference ($P < 0.05$ unpaired, two-sided *t*-test for total OSN). The error bars for the simulation results represent s.d. of five independent simulations.

Source data are available online for this figure.

© 2018 The Authors

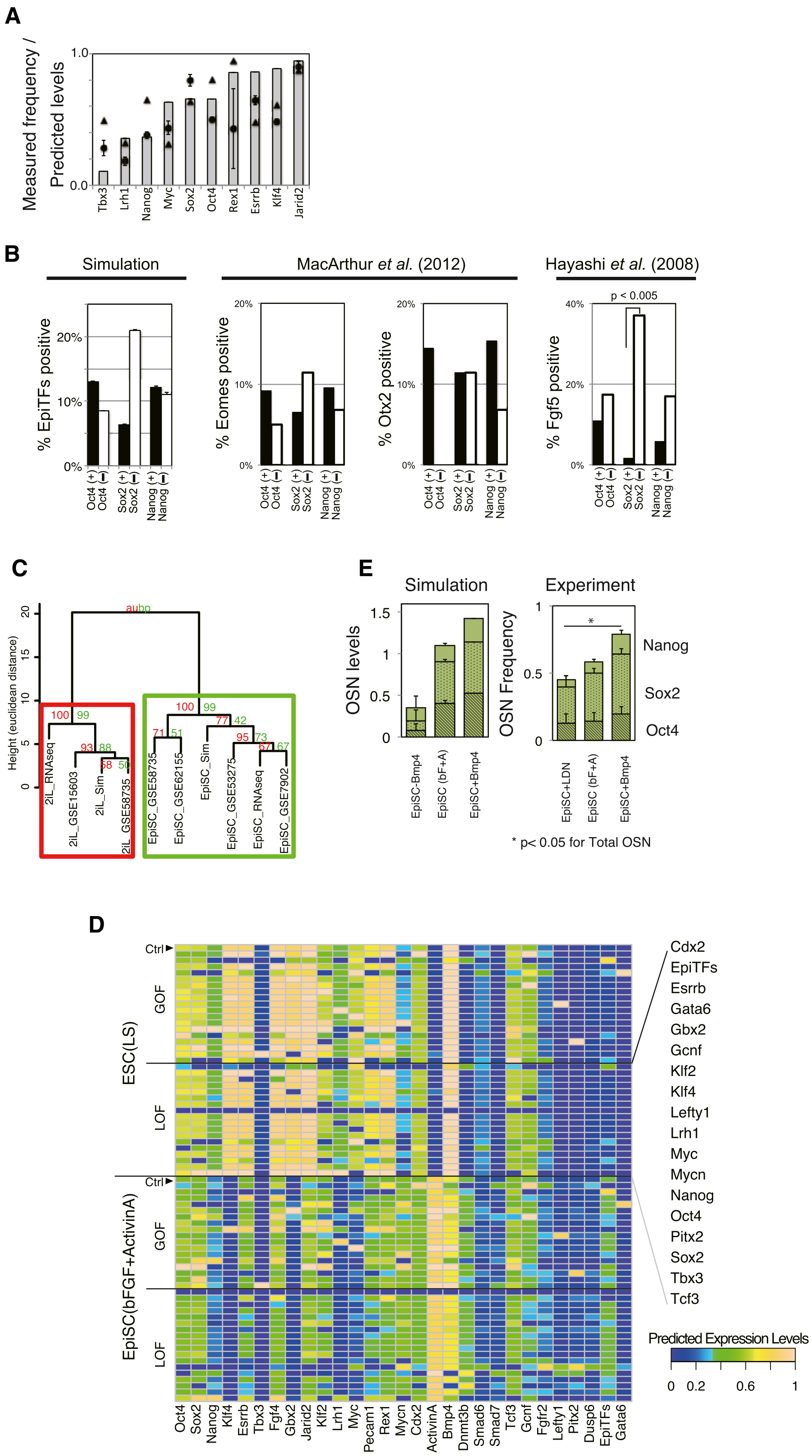

Figure EV3.

 © 2018 The Authors

Figure EV4. LIF stabilizes the pluripotent population while 2i up-regulates OSN; related to Fig 4.

A Summation of frequencies (0–1) of O/S/N-positive cells assessed by high content screening at 2 days after medium replacement ($n = 6$ for Oct4 and Sox2, $n = 4$ for Nanog; the error bars represent s.d.). Asterisk indicates the significant difference for total OSN assessed by unpaired, two-sided student t-test (**$P < 0.05$, *$P < 0.1$).

B Upper panel: A measure of Pearson's correlation (PCC) for OSN in 2i-supplemented conditions, quantified using high content screening ($n = 6$ for Oct4 and Sox2, $n = 4$ for Nanog; the error bars represent s.d.). Lower panel: A measure of PCC for OSN in the results of a minimal perturbation-sensitivity analysis described in (C). The significance was assessed by Wilcoxon exact rank test (*$P < 0.05$, **$P < 0.01$).

C The results of minimal perturbation-sensitivity analysis of the model where the model network was perturbed by removing single regulatory edges. Purple indicates down-regulation of the genes by removing the regulatory edge, which means the regulation had a positive role on the expression level of O/S/N, while green indicates the reverse. Note that the results shown are the effects of regulatory edge removal: the removal of inhibitory regulation from Cdx2 to Oct4 increases Oct4, which means that the regulation edge acts as a negative effector for Oct4 level.

D The difference in PSC population stability between 2iL and 2i−L conditions was assessed via published single gene LOF and double genes LOF *in vitro* (Dunn *et al*, 2014; EXP) and *in silico* (SIM). The upper panel depicts the experimental results for the relative count of alkaline phosphatase (AP) positive cells to untreated colonies upon gene manipulations. The simulation data (lower panel) shows population-averaged expression level of Oct4 relative to controls (2iL and 2i−L) where the manipulated gene was set as continuously OFF. Blue nodes indicate the response in 2i−L, while red nodes indicate those in 2iL ($n = 5$ for simulation and $n = 3$–5 for experimental).

E The experimental inputs (additives of the conditions) corresponding to the simulation inputs shown in Fig 4E. Abbreviations used are as follows: Jaki for Janus kinase (JAK) inhibitor, Dkk1 for Dickkopf1, and Alki for chemical inhibitor selective for Activin receptor-like kinase (ALK) 4/5/7.

F Predicted levels (left) and measured gene-expressing cell frequencies (right) of O/S/N for each condition group (L, LIF; W, WNT). The average value of four distinct signal conditions (±BMP±Activin/Nodal) in each group for each gene was calculated and summed up into an OSN score ($n = 6$ for Oct4 and Sox2, $n = 4$ for Nanog; the error bars represent s.d.). Asterisk indicates the significant difference for total OSN assessed by Wilcoxon exact rank test (**$P < 0.05$, *$P < 0.1$).

G Susceptibility of O/S/N expression levels of PSCs to Activin and BMP signal perturbations was predicted (upper panel) and measured (lower panel). Standard deviation per mean of predicted expression levels or gene-expressing frequencies from immunostaining was calculated as a coefficient of variation for each group (±LIF±WNT) including four distinct signal conditions (±BMP±Activin/Nodal).

H Frequencies of O/S/N-expressing cells in the 19 combinatorial signal conditions with and without serum assessed by immunostaining ($n = 2$). Most conditions were equivalent except for −L–W−B+A and +L−W−B−A. The notable down-regulation of OSN in −L+W+B−A (=2iJ+B−A) was seen in both with- and without-serum conditions. The error bars represent s.d. of five independent simulations.

Source data are available online for this figure.

© 2018 The Authors

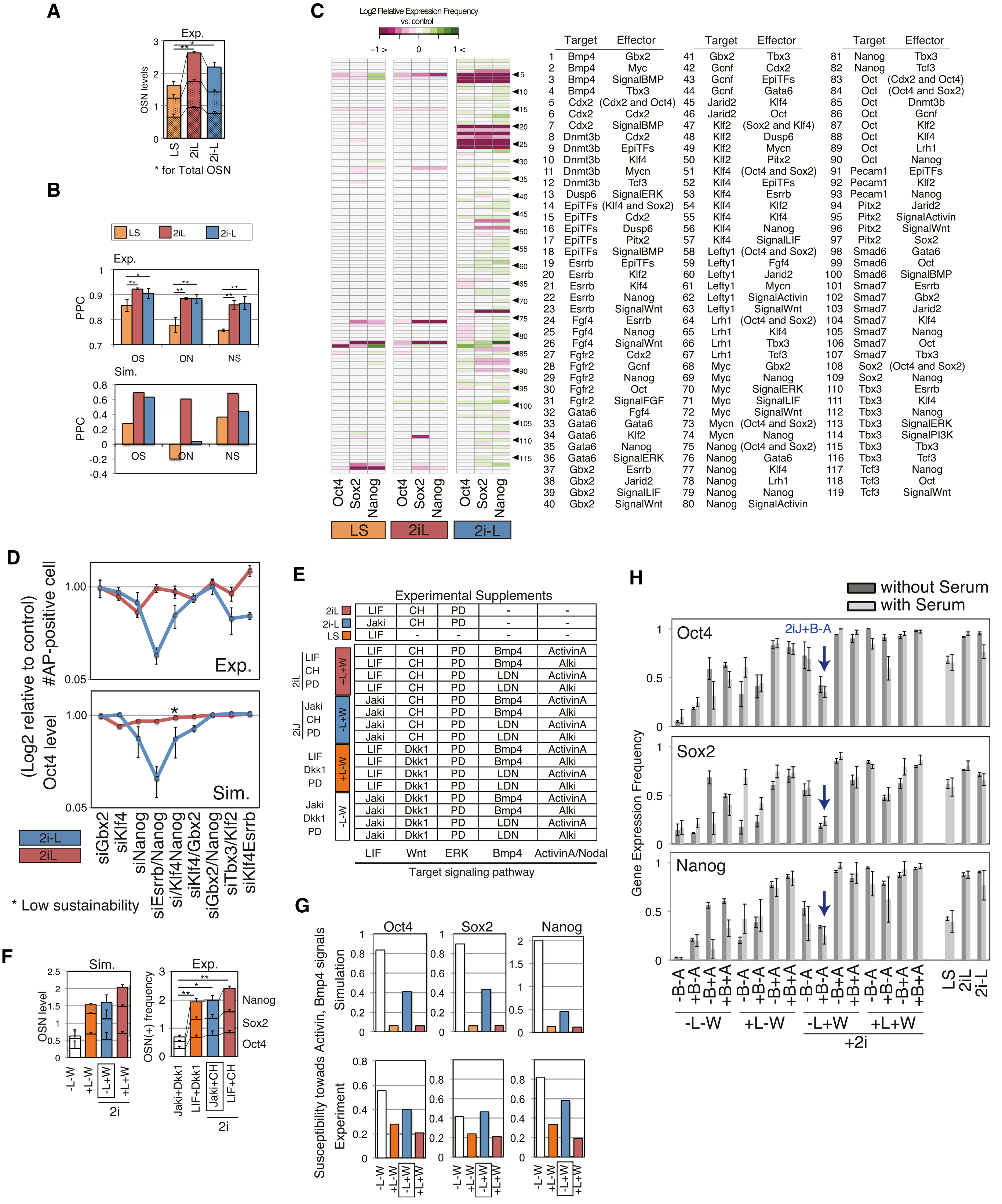

Figure EV4.

 © 2018 The Authors

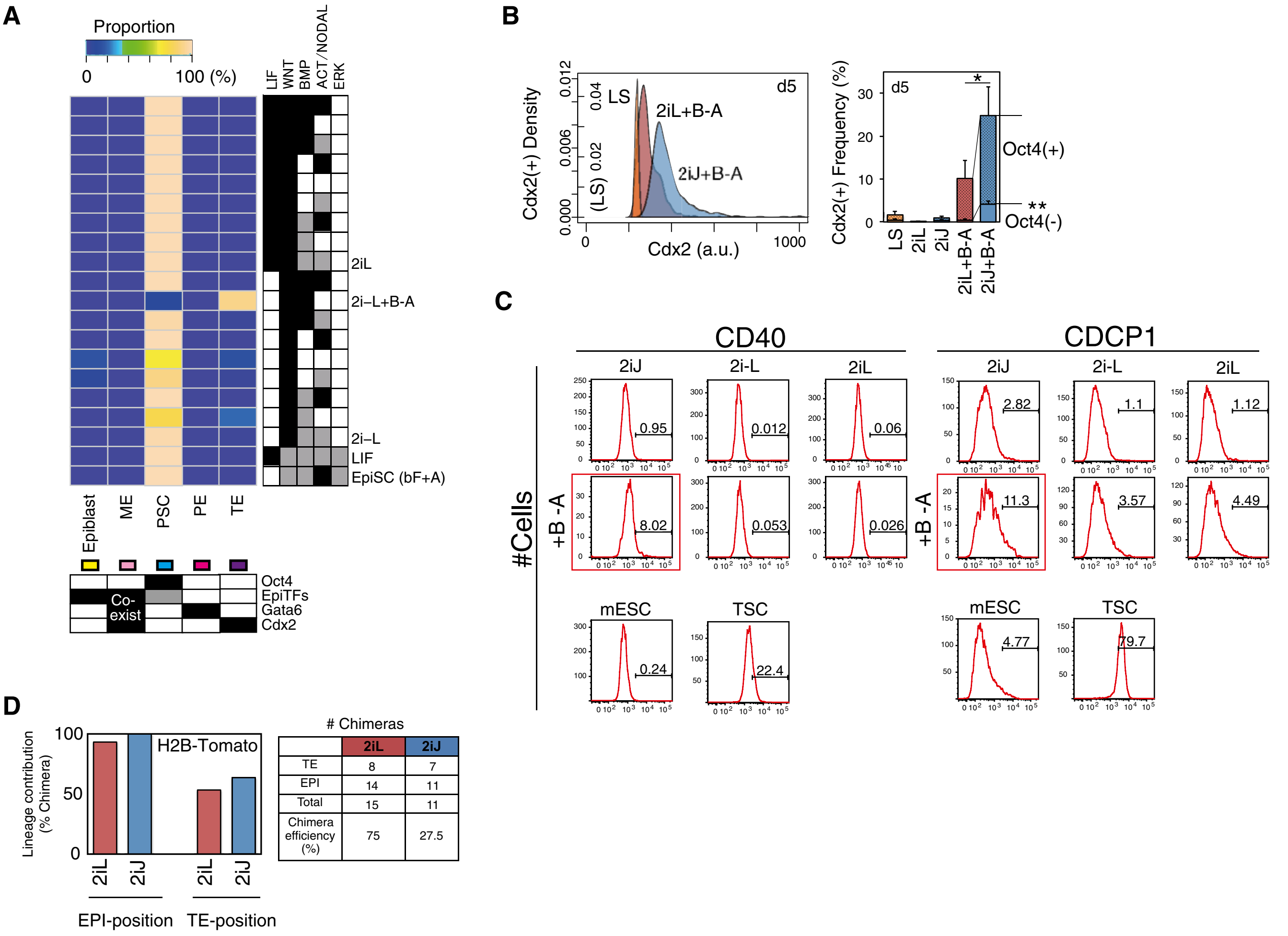

Figure EV5. ***In silico* subpopulation analysis reveals mESCs exit pluripotency toward TE-like lineage in 2iJ+B−A condition; related to Fig 5.**

A *In silico* subpopulation analysis of possible signaling input combinations with 2iL and 2i−L. The threshold values for predicted expression levels of lineage specifiers in each SCC are set as follows: Oct4 = 0.3, EpiTFs = 0.2, and Gata6 = 0.5 and Cdx2 = 0.7.

B Frequency of Cdx2$^+$ population including TE-like subpopulation (Cdx2$^+$/Oct4$^-$) after 5 days in culture measured by immunostaining. Data represent the means and the error bars represent s.d. of six replicates consisting of two biological replicates with three technical replicates in each. The representative density plot of immunostaining of Cdx2 on day 5 is shown in the left panel. The significance was assessed by Wilcoxon exact rank test (*$P < 0.05$, **$P < 0.0005$) for the frequencies of total Cdx2$^+$ and Cdx2$^+$Oct4$^-$ comparing 2iL+B−A and 2iJ+B−A.

C Histograms for expression of TE-enriched cell surface markers, CD40 and CDCP1. Shown are mESCs treated in 2iJ (1st column), 2i−L (2nd column), or 2iL (3rd column), in unsupplemented medium (1st row) or BMP4 and ALKi supplemented medium (+B−A; 2nd row). Control mESCs (kept in control LS) and TSCs are shown at the bottom.

D *In vivo* lineage contribution frequency and chimera efficiency of H2B-Tomato ESCs (Morgani *et al*, 2013) treated with either 2iL or 2iJ in the presence of serum.

Source data are available online for this figure.
